# Deep Learning-Based Fault Diagnosis via Multisensor-Aware Data for Incipient Inter-Turn Short Circuits (ITSC) in Wind Turbine Generators

**DOI:** 10.3390/s25082599

**Published:** 2025-04-20

**Authors:** Qinglong Wang, Shihao Cui, Entuo Li, Jianhua Du, Na Li, Jie Sun

**Affiliations:** 1 Department of Mechanical Engineering, North China Electric Power University, Baoding 071003, China; 13183131206@163.com (S.C.); lientuor@163.com (E.L.); huahuahuahua204@gmail.com (J.D.); leena73@163.com (N.L.); 2 Hebei Engineering Research Center for Advanced Manufacturing & Intelligent Operation and Maintenance of Electric Power Machinery, North China Electric Power University, Baoding 071003, China; 3 The State Key Laboratory of Digital Steel, Northeastern University, Shenyang 110819, China

**Keywords:** wind turbine (WT), inter-turn short-circuit (ITSC) faults, deep learning, fault diagnosis

## Abstract

Wind energy is a vital pillar of modern sustainable power generation, yet wind turbine generators remain vulnerable to incipient inter-turn short-circuit (ITSC) faults in their stator windings. These faults can cause fluctuations in the output voltage, frequency, and power of wind turbines, eventually leading to overheating, equipment damage, and rising maintenance costs if not detected early. Although significant progress has been made in condition monitoring, the current methods still fall short of the robustness required for early fault diagnosis in complex operational settings. To address this gap, this study presents a novel deep learning framework that involves traditional baseline machine-learning algorithms and advanced deep network architectures to diagnose seven distinct ITSC fault types using signals from current, vibration, and axial magnetic flux sensors. Our approach is rigorously evaluated using metrics such as confusion matrices, accuracy, recall, average precision (AP), mean average precision (mAP), hypothesis testing, and feature visualization. The experimental results demonstrate that deep learning models outperform machine learning algorithms in terms of precision and stability, achieving an mAP of 99.25% in fault identification, with three-phase current signals emerging as the most reliable indicator of generator faults compared to vibration and electromagnetic data. It is recommended to combine three-phase current sensors with deep learning frameworks for the precise identification of various types of incipient ITSC faults. This study offers a robust and efficient pipeline for condition monitoring and ITSC fault diagnosis, enabling the intelligent operation of wind turbines and maintenance of their operating states. Ultimately, it contributes to providing a practical way forward in enhancing turbine reliability and lifespan.

## 1. Introduction

### 1.1. WT Technology Development

**Figure 1:**
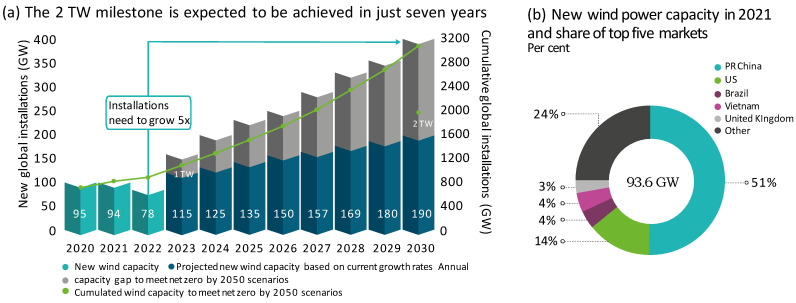
Global installed wind turbine capacity growth trends and forecasts [4,10].

Wind and solar energy have demonstrated technological and cost competitiveness, leading to anticipation of a renewables revolution [1,2,3]. To meet the rising demand for clean energy, the global installed wind power capacity needs to increase almost ten-fold in the next 30 years. According to Figure 1, the global cumulative wind power installations (excluding grid connection) amounted to nearly 940 GW as of the end of 2022, which was primarily propelled by the anticipated rapid expansion of the wind sector in China [4,5]. A wind turbine (WT) system is a complete electromechanical system consisting of various elements that are designed to continuously extract significant quantities of kinetic energy from the wind [6]. The primary objective of WTs is to convert this energy into clean, carbon-free electricity [7]. The global aspiration for clean energy has been the driving force behind the rapidly growing wind power industry. In recent decades, WT manufacturing techniques have made significant progress [8]. Contemporary WTs are larger than ever before, with some exceeding 200 m in size. During the mid-1990s, the rated power of WTs was less than 1000 kW. However, the market for modern WTs is now dominated by multi-megawatt machines, with some as powerful as 10 MW, that are designed primarily for offshore wind energy applications [9].

Although WTs have seen rapid advancements, WT operation and maintenance (O&M) techniques still trail behind those used for steam, hydro, and gas turbines in conventional power plants. Due to the high maintenance costs [11] of offshore wind turbines, the scarcity of suitable construction sites, and the challenges of handling complex wind loads, onshore power generation remains more prevalent than offshore generation. This places wind turbines in more challenging geographic environments [12], such as remote locations with high humidity, salt spray, significant temperature fluctuations, and snow-covered terrains [13]. Additionally, prolonged and large-scale load variations introduce significant uncertainties in the prognostics and health management of WTs. The primary challenge lies in the timely detection of incipient faults and the scheduling of maintenance to reduce the O&M costs of wind farms through condition monitoring and fault diagnosis (CMFD) techniques [14,15]. While CMFD technologies have made significant strides in signal processing and diagnostic methods over the past few decades, including advancements in the time, frequency, and time-frequency domains, intelligent diagnostics, image processing, data fusion, data mining, and expert systems [16], the complexity of wind turbines necessitates the further exploration of CMFD technologies that are tailored specifically for these systems. Thus, CMFD technologies are becoming increasingly critical to maximizing economic efficiency and minimizing downtime in wind turbine operations.

**Figure 2:**
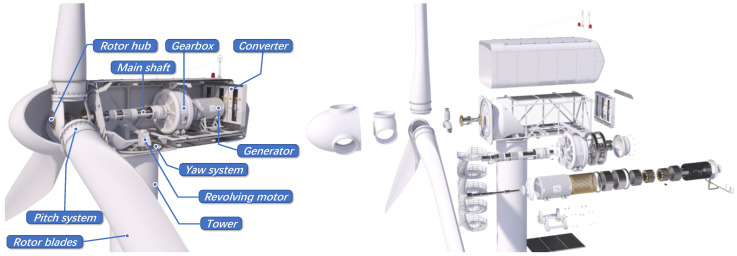
A typical WT with main subsystems and components shown [13,18,19].

As illustrated in Figure 2, a WT is a sophisticated electromechanical system comprising multiple components and subsystems, including the rotor hub, blades, shaft, gearbox, and generator, among others. WT systems are highly complex and comprised of numerous components that operate at high power. As a result, they are prone to frequent failures that can cause long periods of downtime that last several days and result in significant financial losses. Therefore, effective fault detection is critical in maximizing the production capacity of WTs [17].

### 1.2. ITSC Fault Diagnosis for WT Generators

The early fault diagnosis of WT generators is crucial to enhancing the service life of WTs and curtailing the O&M costs of WT systems. Generator failures can be categorized according to their internal and external causes [7]. The internal causes of generator failures are the results of mechanical or electrical faults, including rotor bar breakage, shaft bending, air gap eccentricity, mass imbalance, and bearing failures for mechanical faults, and damaged stator/rotor insulation, electrical imbalance, faulty magnetic circuits, open circuits, and short circuits for electrical faults [18]. Among these faults, inter-turn short-circuit (ITSC) faults are particularly challenging since they are difficult to detect using traditional protection schemes [19]. ITSC faults involve a several-turn short circuit in the one-phase winding, which can lead to critical insulation breakdown due to heating in the shorted turns. If left unattended, ITSC faults may escalate to a phase-to-phase or phase-to-ground short-circuit, resulting in various adverse effects such as unbalanced line currents, vibrations and noises, decreased efficiency, and excessive heating.

In general, the early detection of ITSC faults in WT generators is crucial for preventing catastrophic failures, improving the reliability of WTs, reducing servicing costs, and extending the entire system lifecycle. ITSC fault diagnosis involves measuring various factors, such as generator voltages and currents, the moment, the vibration, the temperature, and otoacoustic emissions, for the purpose of fault analysis. This contributes to the assessment of the technical status of the machine by extracting damage symptoms from the detected diagnostic signals. It is imperative to develop effective and efficient methods for detecting ITSC faults in WT generators. Such methods would help mitigate the potential damage and expenses associated with generator failures. By accurately assessing the condition of the machine, operators can identify and fix faults before they lead to more severe problems.

Transducers are commonly deployed on generators to acquire important operating information, such as voltages, currents, and vibrations, during their operation [20]. The signals from these sensors are then used to extract specific features that can be applied to differentiate between faulty and normal situations. The accurate extraction of fault symptoms is crucial for estimating the condition of the machine and indicating possible damage. The analysis is then applied to the sensor measurement signals to see how the signals change with the time, frequency, or time-frequency. However, efforts to fully automate traditional analysis methods often fail, and the diagnostic process can be cumbersome, requiring long fault diagnosis times and manual interpretation. Advancements in artificial intelligence technology have led to the development of data-driven and machine learning-based methods that hold great potential in fault detection [21,22]. To surmount the limitations of traditional methods, intelligent ITSC fault diagnosis systems can be developed. These systems generally require the application of data-driven methods, including deep neural network (DNN) models, support vector machine (SVM) [23,24,25] models, random forests (RFs) [21,26,27], naive Bayes models, k-nearest neighbor (k-NN) [28] models, and expert-based systems [7].

In recent years, deep learning methods have been developed and applied in the field of fault diagnosis [29,30,31,32]. Deep learning has shown promising results in extracting representative features from original signals by utilizing its in-depth architecture. Compared to shallow models, utilizing deep structures to learn feature representations is advantageous, as the highly semantic information learned through deep structures is more impressive than the scattered and poor representations learned through extensive shallow models. The deepness of the system allows for better feature learning, while the batch normalization (BN) technique encourages faster convergence, which can lead to improved results. Moreover, deep structures provide great domain adaptability, while the simple architecture of shallow structures is not conducive to domain adaptability, effective representation, or sensitivity to new data. Therefore, deep learning has been widely used in various fields, such as computer vision, speech recognition, biomedicine, and natural language processing, among others [33,34,35].

Given the outstanding performance of deep learning [36], particularly DNN models, in feature learning, researchers have become increasingly interested in exploring the application of deep learning models in WT fault diagnosis [37]. Attallah et al. (2023) [7] used infrared thermography for rotor internal short-circuit fault diagnosis and monitoring techniques for offshore WTs and discussed fault diagnosis methods based on deep convolutional network (DCNN) models. Lei, Liu, & Jiang, (2019) [38] proposed a fault diagnosis framework for WTs that uses an end-to-end LSTM model to learn features directly from time-series data and capture long-term dependencies, and which outperforms traditional methods in fault classification and demonstrates robustness on small datasets. Cherif et al. (2020) [39] developed an ANN-based method for the early detection of ITSC faults in the stator winding of induction motors, using a novel indicator based on the discrete wavelet energy ratio (DWER) of three stator currents. Kumar & Hati (2021) [40] discussed the adoption of a deep convolutional neural network (DCNN) with an adaptive gradient optimizer for bearing and rotor fault detection in squirrel-cage induction motors (SCIMs), employing sensor data fusion in their model training and testing. Xue, Xiahou, Li, Ji, & Wu, (2019) [41] introduced a data-driven fault diagnosis method, utilizing a long short-term memory network, to detect multiple open-circuit switch faults of the back-to-back converters in doubly fed induction generator-based WT systems, and demonstrated its effectiveness through simulations and experimental tests. Dhibi, Mansouri, Bouzrara, Nounou, & Nounou (2022) [42] focused on developing and validating an effective neural network-based ensemble approach for fault detection and diagnosis in wind energy conversion systems, using techniques such as bagging, boosting, and random subspace combination. Bazan et al. (2017) [43] presented a pattern recognition method using mutual information between phase current signals to detect stator winding short circuits in three-phase induction motors, achieving high classification accuracies even under varying load torque and power supply voltage conditions. Li et al. (2024) [44] present a partial domain adaptation method using deep adversarial learning to address the challenge of limited target-domain data for making accurate cross-domain RUL predictions in machinery. Zhang et al. (2024) [45] present a Swin transformer-based method for the accurate SOC prediction of lithium-ion batteries, addressing data noise to enhance electric aircraft safety. Chen et al. (2024) [46] propose a dynamic vision-based spiking neural network for contactless cross-domain fault diagnosis that does not require target-domain data. In two studies, Gao et al. (2023–2024) [47,48] advanced the field by proposing a domain feature decoupling network (DFDN) that decomposes and adaptively fuses domain- and fault-related features to enable the zero-shot fault diagnosis of rotating machinery under unseen operating conditions and a complex convolutional self-attention autoencoder (CCSAE) that leverages self-attention-enhanced complex feature representations to achieve highly accurate early fault detection in analog circuits.

It can be deduced that, despite efforts to detect ITSC faults in wind turbines, this technology has not yet been fully developed. With the rapid expansion of wind power capacity, the identification of these faults remains a significant challenge for engineers, requiring ongoing efforts to address it. To date, there has been a lack of systematic analyses of the effectiveness of deep learning techniques, specifically deep convolutional networks, in comparison to traditional machine learning algorithms in detecting ITSC faults. This underscores the importance of continual efforts to simplify the ITSC fault detection process and achieve comprehensive fault detection. Simultaneously, enhancing the performance of fault diagnosis models is crucial for establishing an efficient, accurate, reliable, and robust fault detection and identification system. The proposed deep learning-based framework directly addresses these limitations by leveraging the powerful feature extraction capabilities of deep neural networks (DNNs). Unlike traditional methods, which may require extensive expert knowledge and manual intervention, our approach automates the fault detection process and can identify subtle fault signatures that would otherwise go unnoticed. The primary advantage of our method lies in its ability to process and analyze complex multi-sensor data, specifically current, vibration, and axial magnetic flux signals, enabling more accurate and timely fault detection. By systematically comparing the performance of our deep learning models to that of traditional machine learning algorithms, we demonstrate a significant improvement in detection accuracy and robustness. Our approach not only detects ITSC faults more reliably but also offers a transferable diagnostic framework that can be adapted to various scenarios of fault detection in wind turbine generators.

Thus, the objective of this study is to explore the potential of data-driven and deep learning techniques for diagnosing ITSC faults in wind turbines and to develop a deep learning-based approach that can accurately identify various categories of short-circuit faults in wind turbine generators without requiring extensive expert knowledge. The primary contributions and findings of this study are as follows:

- This study proposes a transferable deep learning-centered approach for fault diagnosis in wind turbines, offering a novel pipeline encompassing dataset acquisition, model construction, training strategies, and comprehensive evaluation methods;
- The FCNet-5 model achieves a state-of-the-art mAP score of 99.25%, demonstrating superior stability and precision, particularly in challenging fault scenarios;
- The use of three-phase current signals is more effective than using vibration and electromagnetic signals in monitoring generator faults. Differentiating between HI-1 and LI-1 faults, which have small feature intervals, remains challenging. However, deep learning models demonstrate superior robustness in accurately diagnosing these challenging faults using both current and vibration/electromagnetic signals.

The remainder of this paper is organized as follows. Section 2 systematically explains the principle and construction of the wind turbine test bench used herein, as well as the related dataset. Subsequently, various deep learning methodologies are studied, and different architectures of deep network models are designed to achieve state-of-the-art performance in Section 3. Section 4 and Section 5 provide details on model evaluation methods and metrics, as well as the implementation of the deep learning model. In Section 6, the fault recognition performances of the machine learning and deep learning models are thoroughly discussed and compared. Finally, Section 7 presents conclusive summaries.

## 2. WT Test-Bench and Dataset Composition

**Figure 3:**
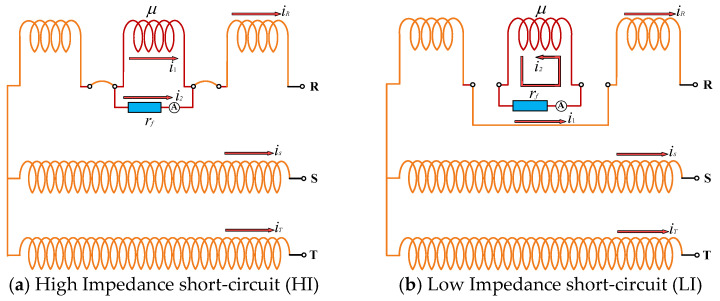
Electrical schematic of the inter-turn short-circuit simulation for the stator R-phase winding [49].

A physics-based simulation is commonly employed to emulate ITSC faults in WTs, which offers the advantage of avoiding substantial mechanical expenses and uncontrollable fault escalation. As illustrated in Figure 3, the principle of a manually simulated short-circuit fault in the R-phase of the stator windings for a star-connected three-phase induction generator is depicted. Different severities of short-circuit faults can be emulated by adjusting the parallel fault loop between the shorted turns [19]. Most incipient short-circuit faults are obtained through a high impedance (HI) short-circuit, as shown in Figure 3a, after which the resistance *r_f_* in the parallel path created for the electric current to flow through is decayed until it reaches the low impedance (LI) case in Figure 3b. The LI state is the state before total degradation, whereby a given quantity of turns is in a fully shorted state. The fully shorted state described above is simulated by removing the coil turns from the circuit but keeping them submerged in the electromagnetic field. Therefore, the severity and level of faults can be controlled by the percentage of shorted coil turns in the R-phase winding μ and the resistance that generates the HI and LI fault loops *r_f_*. It is important to note that all simulated faults are incipient, which implies that the current in the short-circuit fault loop is limited to the rated value. In reality, however, the current is likely to exceed the rated value.

**Figure 4:**
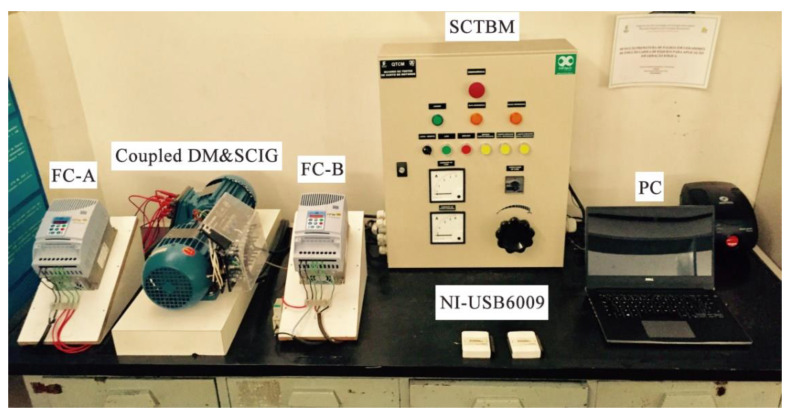
Experimental bench of the stator winding short-circuit fault for WT generator [50].

Many investigators have utilized experimental platforms to study the conversion behavior of artificially created ITSC faults of coil turns in WTs based on the common principles depicted in Figure 3. In this study, we adopt the WT fault simulation test bench and sensor placement strategy established by Xu et al. (2021) [50] and its subsequent work for consistency and clarity. To provide a brief description of the equipment and data acquisition process that we used to build the WT short circuit database (https://github.com/lapisco/Wind_turbine_failure_prediction (accessed on 16 April 2025)), we outline the details here. Note that we utilize the previously referred to dataset in the pre-processing stage in our current study. As illustrated in Figure 4, two mechanically coupled and identical induction machines, which are full-scale and have a fully variable speed, that is, the machines can generate electricity over the entire speed range, and that are rated at 220 V, 60 Hz, 4-pole, 1 hp, 3 A, are used. One machine functions as a drive induction motor (DM) with a frequency-adjustable power supply, which is obtained using a frequency converter (FC-A) to emulate the variable speed wind that drives the rotation of WT blades in a natural wind field. The other machine serves as a squirrel-cage induction generator (SCIG), while a frequency converter (FC-B) feeds its stator windings. When the frequency set in FC-A is higher than that in FC-B, the kinetic energy of the variable speed DC is converted into electrical energy and transmitted to the DC bus of FC-2 via the SCIG. Therefore, an experimental device consisting of FC-A and FC-B, together with coupled DM and SCIG, can emulate the conversion of the kinetic energy of a WT’s blades into electrical energy.

**Figure 5:**
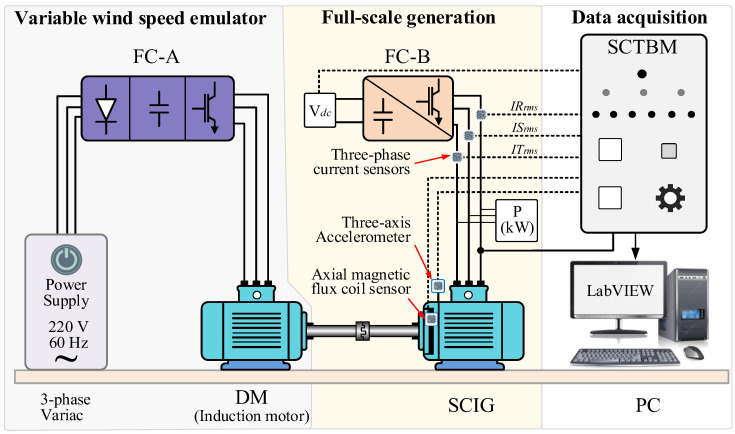
Schematic of the principle and sensor layout of the WT test bench.

In addition, a short-circuit test board with a data acquisition and transmission module (SCTBM) is utilized to implement the short-circuit loop setting for the R-phase turns of the stator winding in the SCIG. In addition, a short-circuit test board with a data acquisition and transmission module (SCTBM) is utilized to implement the short-circuit loop setting for the R-phase turns of the stator winding in the SCIG. A data acquisition instrument (the NI-USB6009 module programmed to read signals for 10 s with a sampling frequency of 5 kHz and a resolution of 14 bits) is integrated into the SCTBM to acquire signals such as the current, voltage, and frequency. The test bench’s device and electrical principles, along with the sensor arrangement, are illustrated in Figure 5. Different sensors are positioned on the SCIG, including a three-phase current, a three-axis accelerometer, and an axial magnetic coil detector. Additionally, the frequency (*f_g_*) and the DC bus voltage (*V*_dc_) at the SCIG side are recorded in the data acquisition system, with the former varying from 43.65 Hz to 59.27 Hz and the latter from 210 V to 380 V.

According to the principles of the short-circuit fault depicted in Figure 3, the percentage of shorted coil turns in the stator R-phase winding (μ) is set to 1.43%, 4.81%, and 9.26%, respectively, through the SCTBM module. Combined with the HI and LI fault settings in Figure 3, six types of short-circuit faults are emulated, respectively designated as ‘HI-1’ (1.43%), ‘HI-2’ (4.81%), ‘LI-3’ (9.26%), ‘LI-1’ (1.43%), ‘LI-2’ (4.81%), and ‘LI-3’ (9.26%). These faults evolve sequentially from the initial stage (HI-1) to the severe situation (LI-3), but are also incipient for WTs. A database of 1356 acquisitions, consisting of the six short-circuit faults and normal conditions, is obtained from the three-phase current sensor and named SENSORC. An additional database is obtained by the tri-axial vibration sensor and the axial magnetic flux sensor, with a total of 1325 samples, and denoted as SENSORA_SENSORV. This dataset setup allows for comparing and evaluating the difficulty of diagnosing incipient short-circuit faults through different sensors and the robustness requirements for the learning model, enabling engineers to choose the most suitable sensor arrangement for monitoring the health status of actual WTs.

To construct the relevant dataset, three widely recognized feature extraction techniques, fast Fourier transformation (FFT), higher-order statistics (HOS), and a structural co-occurrence matrix (SCM), are applied. The composition of the datasets, including the extracted features, is thoroughly described in Table 1.

## 3. Related Methodology and Network Architecture

### 3.1. Basic Components of the Network Model

Deep convolutional neural networks (DCNNs) are powerful models that use multiple convolutional layers and nonlinear operations to automatically extract features from input data and transform them into high-level representations [11]. Due to employing hierarchical feature learning, DCNNs have an enhanced ability to capture complex patterns and support end-to-end learning.

**Figure 6:**
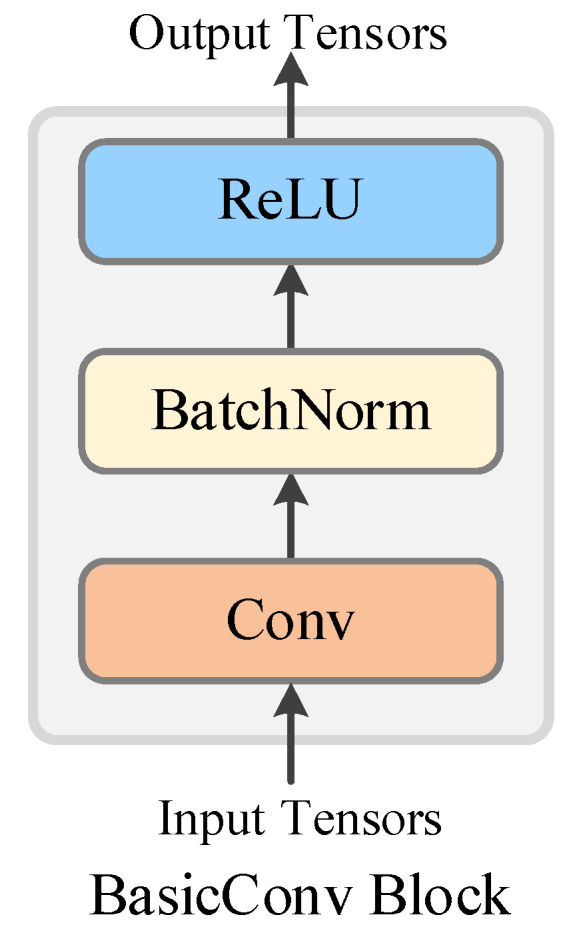
Basic convolutional block in the DCNN model.

In the event of changes in input features to a neural network’s learnable parameter layer, the parameters must be relearned, a challenge known as internal covariate shift. To address this, we apply batch normalization (BN) to standardize the input distribution of each layer, enhancing scale invariance. Our basic convolutional layer structure thus includes convolution, batch normalization, and activation, as illustrated in Figure 6.

We employ the cross-entropy loss function as the criterion of the network to calculate the variance between the predicted fault types and the actual fault types; the cross-entropy loss function is:

(1) $$\begin{array}{l} L_{\mathrm{cross}\text{-}\mathrm{entropy}}=-\frac{1}{N}\sum_{i=1}^{N}\sum_{c=1}^{C}t_{c}^{\left(n\right)}\log{\hat{y}}_{c}^{\left(n\right)}\\ \mathrm{where}\;{\hat{y}}_{c}^{\left(n\right)}=\frac{e^{f_{c}(x^{\left(n\right)};w_{c})}}{\sum_{j=1}^{C}e^{f_{j}(x^{\left(n\right)};w_{j})}} \end{array}$$

where *C* is the number of types of short-circuit faults, $x^{\left(n\right)}$ is the *n*-th sample, $f_{c}\left(∙\right)$ is the output value of the network model for the real *c*-th fault, $w_{c}$ is the weights to be learned for the network model, ${\hat{y}}_{c}^{\left(n\right)}$ is the posterior probability of the model output of sample $x^{\left(n\right)}$ in each fault class, and $t_{c}^{\left(n\right)}$ is the one-hot vector used to encode the fault category labels.

### 3.2. Residual Learning and Related Network Architectures

General DCNN models typically use stacked convolutional layers to extract features from input data. However, as the network depth deepens to a certain level, both training and testing errors can become progressively worse. This phenomenon is known as the degradation problem, and means that increasing the depth of the network may not necessarily lead to better performance. Surprisingly, the degradation problem is not caused by vanishing or exploding gradients. To address this issue, He, Zhang, Ren, & Sun (2016) [51] developed the residual network (ResNet), which proposes residual learning, allowing data information to be directly passed across learnable layers using residual blocks. The residual block consists of two convolutional layers and a skip connection, which enables information to be passed directly to subsequent layers, thereby facilitating the learning of an identity map and improving the model’s accuracy. The simple structure of the residual block also makes it easy to train. Moreover, stacking multiple residual blocks enables the construction of deeper networks, resulting in better performance.

**Figure 7:**
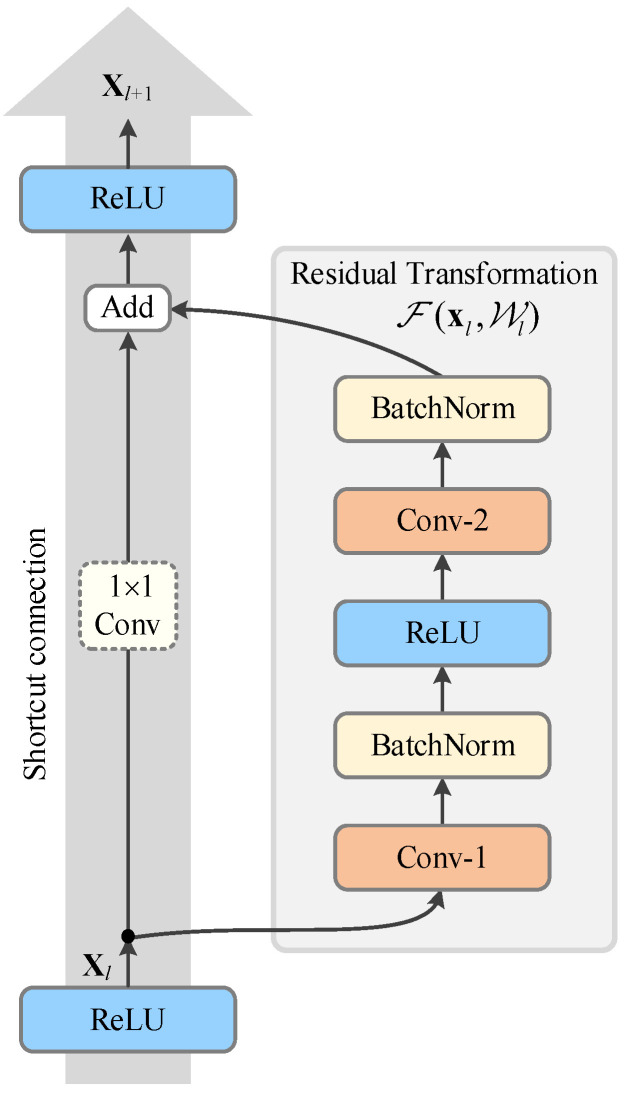
A residual learning block (ResBlock) in the convolutional neural network.

The residual blocks in the network propagate the feedforward information by iterating over the general form of the following equation:

(2) $$\begin{array}{c} F(x_{l},W_{l})=H{\left(x\right)}_{l}-x_{l}\\ z_{l}=H\left(x_{l}\right)=x_{l}+\left(H{\left(x\right)}_{l}-x_{l}\right),\\ x_{l+1}=f\left(z_{l}\right), \end{array}$$

where $x_{l}$, $W_{l}$, $z_{l}$, and $x_{l+1}$ are the input, weight parameters, net activation, and output of the *l*-th layer, respectively, H is the new feature transformation function that the stacked layer needs to learn, $F(x_{l},W_{l})$ is the residual mapping to be learned, and f is a nonlinear activation function. Equation (3) is implemented in the network by the residual block, as shown in Figure 7. If the input tensor needs to be dimensioned down in this residual block, we add an affine transform to the branch of the skip connection and implement it with the 1 × 1 convolutions.

**Figure 8:**
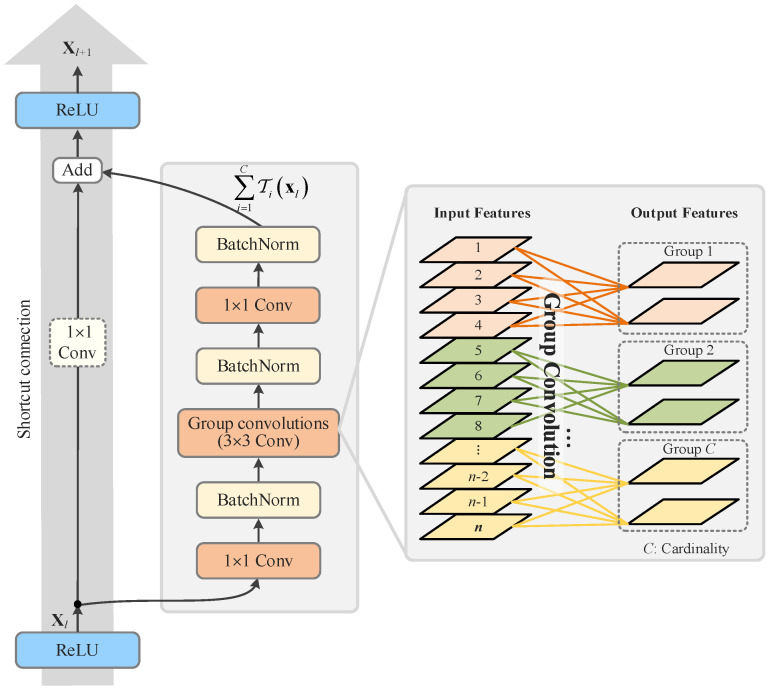
Aggregated residual transformation based on the idea of group convolution and cardinality in the ResNeXt.

ResNeXt is a kind of deep network architecture in which the thoughts of deep residual learning and group convolution are combined to enhance the representational capability of the network [52]. The basic structure of ResNeXt comprises several residual blocks, each of which contains multiple grouped convolution units and one skip connection. The creation of a group convolution unit involves grouping the input feature maps by channel, performing convolution operations within each group, and then merging the output of each group to obtain the final output feature map. This process is referred to as the split-transform-merge process, and the number of groups is determined by the concept of cardinality. By using the split-transform-merge process, the number of model parameters and computations can be reduced, thereby improving the model’s efficiency. The equation given in Equation (2) can be expressed as follows:

(3) $$\begin{array}{c} F(x_{l},W_{l})=\sum_{i=1}^{C}T_{i}\left(x_{l}\right)\\ z_{l}=H\left(x_{l}\right)=x_{l}+F(x_{l},W_{l}),\\ x_{l+1}=f\left(z_{l}\right), \end{array}$$

where $T_{i}$ is arbitrary residual transformation and *C* is the Cardinality. The residual learning block that we use is then further refined to the structure shown in Figure 8.

**Figure 9:**
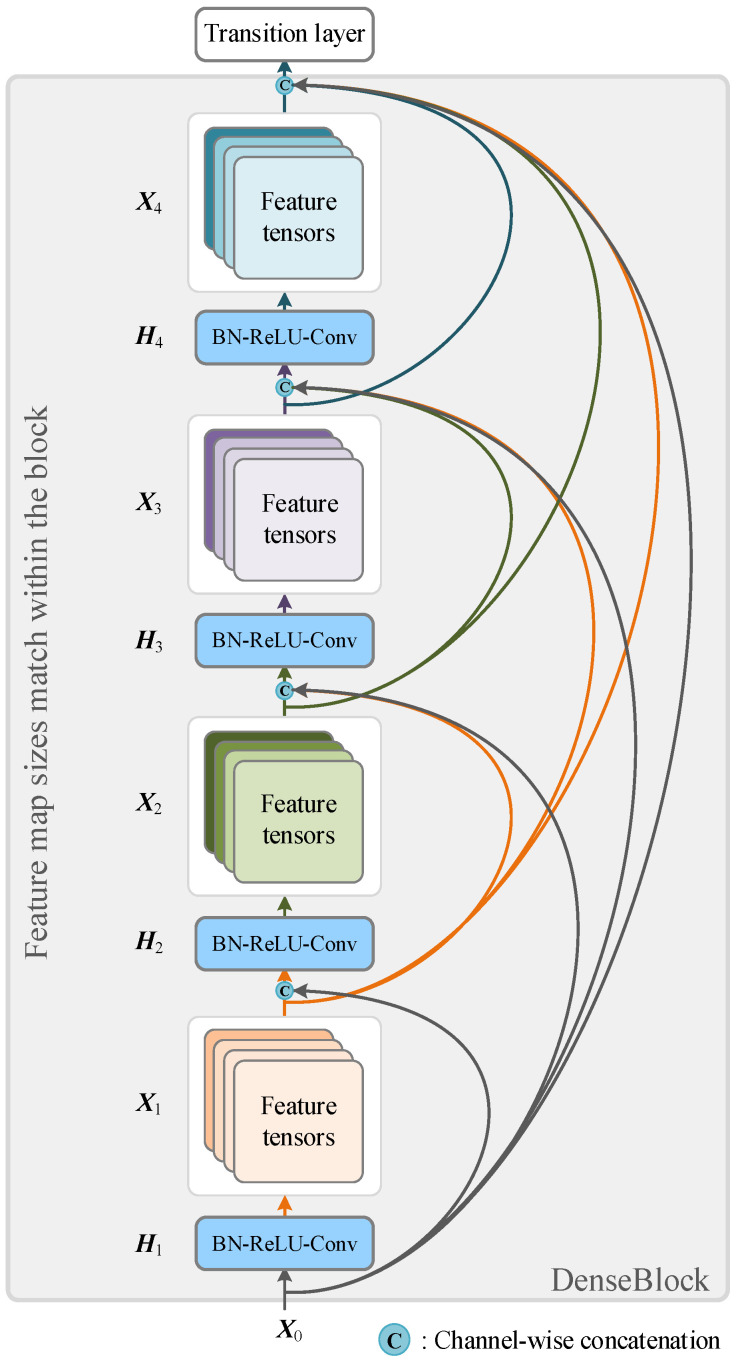
The structure of DenseBlock used by DenseNet ($H_{l}(\cdot )$ represents a non-linear transformation process).

DenseNet is a deep network architecture that is derived from the concept of residual learning and skip connection, with the primary principle of creating dense connections between different layers to take advantage of the feature representations from previous layers [53]. In DenseNet, each layer receives the feature maps from all of the preceding layers and outputs its own feature maps to all of the subsequent layers. The dense connections help to retain more feature information and enhance the backpropagation of the gradient, resulting in easier training of the network. Unlike the residual block in ResNet, the basic building block in DenseNet is the dense block, which contains multiple layers that connect to all previous layers and concatenate their feature maps. In addition, to reduce the output size of the dense block, a transition layer is introduced in DenseNet, which comprises a convolutional layer and a pooling layer that control the number of parameters in the model while reducing the size of the feature map. Figure 9 shows the DenseBlock we built for stacking to construct a DenseNet.

**Figure 10:**
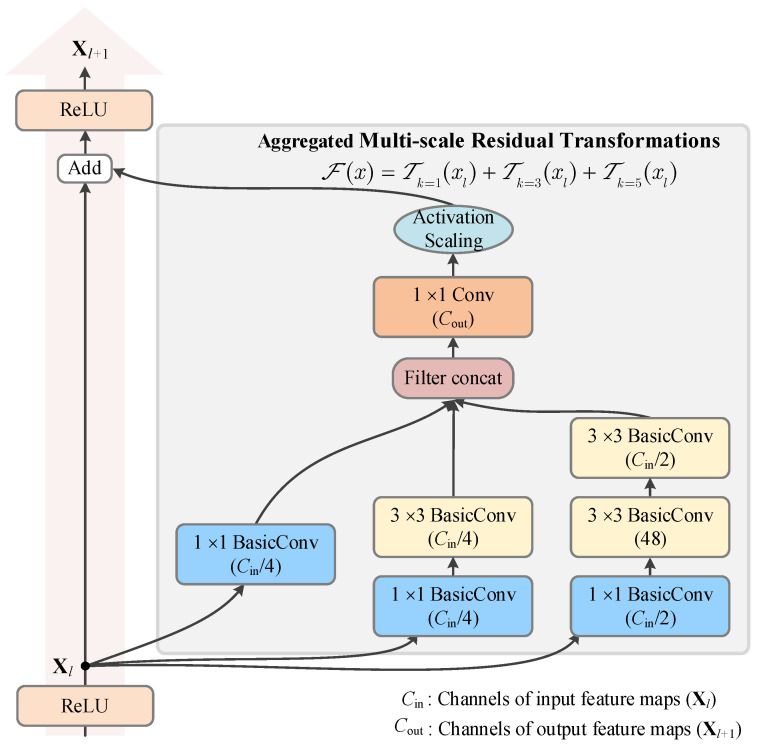
The interior grid module of the inception block with shortcut connection.

In addition, we have constructed VGG [54] and Inception-ResNet [55] architectures for short-circuit fault diagnosis. The VGG network architecture utilizes multiple small 3 × 3 convolutional kernels instead of one large kernel, which enhances the depth and nonlinear capability of the network. On the other hand, the Inception-ResNet architecture combines the design of a local network topology and residual learning to enhance the network’s performance without increasing the model’s complexity. The key aspect of this architecture is the aggregation of multi-scale residual transformations in the inception module that is carried out by incorporating different-sized convolutional kernels in different branches. In Figure 10, we illustrate the inception module designed for short-circuit fault diagnosis in WTs.

### 3.3. Network Depth and Baseline Machine Learning Algorithms

Based on the methodology of deep learning that is detailed above, we constructed different DCNN models and named them according to their number of layers of learnable parameters, i.e., ResNet-31, ResNeXt-22, DenseNet-37, VggNet-18, and Inception-ResNet-59, respectively. The detailed architecture of these models is provided in Appendix B. Since the data samples of short-circuit faults of WTs are one-dimensional tensors, all network models employ one-dimensional convolution. Furthermore, we developed a fully connected network with five learnable parameter layers and named it FCNet-5. The depths of the networks were determined experimentally, and each network structure achieved optimal generalization performance at that depth. Additionally, when deploying deep learning models in industrial sites, there are strict requirements regarding the computational power and size of the model. Thus, we introduced two metrics, namely FLOPs (floating-point operations), which measure the algorithm/model’s complexity, and Params (magnitude of the model parameters), which identify the model’s size. These two metrics reflect the performance requirements for the hardware that is used, such as the GPU and the amount of the machine’s memory that the model uses. Table 2 provides the FLOPs and Params for each network. The detailed structural diagrams and the feedforward computation procedures of the network models are given in Appendix B.

For comparison to deep learning models, we also constructed six traditional machine learning algorithms for short-circuit fault diagnosis based on support vector machine (SVM), naive Bayes, K-nearest neighbor (KNN), and random forest models, with the kernel functions of the SVM employing a linear function (SVM-LINEAR), a radial basis function (SVM-RBF), and a polynomial function (SVM-POLY), respectively.

## 4. Model Evaluation and Selection

### 4.1. Evaluation Metrics

The confusion matrix is a valuable tool for assessing the performance of a classification model and is primarily used to compare the model’s classification results with the actual information of the instances. Each row of the confusion matrix corresponds to the true fault class of the instance, while each column indicates the predicted fault class of the instance. As shown in Table 3, we have assigned numerical codes from 0 to 6 for the seven fault categories, ranging from Normal to LI-3. Specifically, the entries in the confusion matrix ***C*** are defined such that *C_i,j_* denotes the number of instances that are known to belong to fault class *i* and the number that are predicted to belong to fault class *j*.

In addition, it is crucial for the models to diagnose all fault situations with the lowest possible occurrence of fault-missing diagnosis behaviors, as this is a fundamental requirement for the models’ use by engineers. If we consider all fault types as negative instances and the normal categories as positive instances, Table 4 can be simplified into a binary classification task. All instances can be classified into four categories—true positive, false positive, true negative, and false negative—based on the combination of real fault categories and predicted fault categories, and their corresponding samples are represented by *TP*, *FP*, *TN*, and *FN*, respectively. Therefore, the confusion matrix for the binary classification task was simplified, as shown in Table 4.

The sum of the *TP*, *FP*, *TN*, and *FN* instances, together, represents the total number of samples. The three evaluation metrics, Precision%, Recall%, and Specificity%, are defined by the following equation:

(4) $$\begin{array}{l} \mathrm{Precision}\%=\frac{TP}{TP+FP}\times 100\%,\\ \mathrm{Recall}\%\left(\mathrm{Sensitivity}\%\right)=\frac{TP}{TP+FN}\times 100\%,\\ \mathrm{Specificity}\%=\frac{TN}{TN+FP}\times 100\%. \end{array}$$

The best values for Precision%, Recall%, and Specificity% are 100%, while the worst values are 0%. Intuitively, Precision% assesses the model’s capacity to correctly identify positive samples, while Recall% evaluates its capacity to find all positive samples. However, Precision% and Recall% can be contradictory metrics, meaning that, when one is high, then the other tends to be low.

If ${\hat{y}}_{i}$ is the predicted fault class of the *i*-th sample and $y_{i}$ is the corresponding true fault class, then the fraction of correct predictions over $n_{\mathrm{samples}}$ is defined as follows:

(5) $$\mathrm{ACC}\%=\mathrm{accuracy}(y,\;\hat{y})=\frac{1}{n_{samples}}\sum_{i=0}^{n_{samples-1}}1_{Y}({\hat{y}}_{i}=y_{i})\times 100\%,$$

where $1_{Y}\left(∙\right)$ is the indicator function. In an effort to prevent inflated performance estimates on unbalanced datasets, we also employed the metric of balanced accuracy (BlnACC%) [22,56,57,58]. This metric is determined by macro-averaging the recall scores per fault class or, equivalently, the raw accuracy, where each sample’s weight is determined by the inverse prevalence of its true fault class. Thus, on balanced datasets, the BlnACC% equals the ACC%. If $w_{i}$ is the weight of the *i*-th sample $y_{i}$, then we adjust the sample weight as follows:

(6) $${\hat{w}}_{i}=\frac{w_{i}}{\sum_{j}1_{Y}(y_{j}=y_{i})w_{j}}$$

where $1_{Y}\left(∙\right)$ is the indicator function and BlnACC% is defined as follows:

(7) $$\mathrm{BlnACC}\%(y,\hat{y},w)=\frac{1}{\sum {\hat{w}}_{i}}\sum_{i}1_{Y}({\hat{y}}_{i}=y_{i}){\hat{w}}_{i}\times 100\%$$

To evaluate a good model with superior performance, we introduced the composite evaluation metric, average precision (AP%), for each fault-class prediction result. A precision–recall curve (PR curve) was defined from the ground truth fault label and, by varying a decision threshold, we generated a score for the model. The AP% for each fault class was determined by calculating the area under the PR curve, which represents the average precision value of the recall from 0 to 1. This area can be calculated by integration and will not be larger than 1. The greater the area under the PR curve, the better the performance of the model. The AP% for a given fault class is then defined by the following equation:

(8) $$\mathrm{AP}\%=\sum_{n}\left(R_{n}-R_{n-1}\right)P_{n}\times 100\%,$$

where $P_{n}$ and $R_{n}$ are the precision and recall at the *n*-th threshold. Furthermore, to evaluate the performance of the model over the entire dataset in aggregate, we defined the mean average precision (mAP) as the arithmetic average of the AP% of all fault classes. A superior model with good performance will exhibit high Precision% and an increased Recall% as well. In a multi-class fault diagnosis model, mAP represents the mean average precision of all categories. The mAP metric can provide a more comprehensive evaluation of the entire multi-class fault diagnosis model, as it considers not only the overall precision and recall but also the AP value of each category. This is particularly useful in addressing imbalances in the number of samples between different categories or differences in difficulty levels and can provide a more comprehensive assessment of models’ performance. In practical applications, using the mAP as an evaluation metric for a multi-class fault diagnosis model enhances the ability to evaluate the quality of the model and improve its robustness and generalization performance.

### 4.2. High-Dimensional Data Visualization

For a multiclassification task, it is important to visualize the multidimensional outputs obtained from the model’s learning. This enables us to study the sensitivity, interference resistance, and robustness between different models for the given multiclassification task. By doing so, we can select the most suitable model and assess its learning capability using the model itself through the visualization of the high-dimensional model output.

t-SNE (t-distributed stochastic neighbor embedding) [59,60] is a nonlinear dimensionality-reduction algorithm used for visualizing high-dimensional data. The algorithm constructs a probability distribution over pairs of high-dimensional objects in such a way that similar objects have a high probability of being chosen and dissimilar points have an extremely low probability of being chosen. This is done by mapping the proximity relationships of data points to corresponding probability distributions through affine transformations. The final mapping of high-dimensional data into two- or three-dimensional space allows the relative distance between data points to be maintained in both high- and low-dimensional space, providing a clearer expression of the structure and characteristics of high-dimensional data through visualization.

By utilizing the t-SNE technique to map the high-dimensional feature data output by deep learning models to low-dimensional space and by clustering these features, we can visualize how the model responds to various types of noise and disturbances in the input space. For instance, we can visualize the response of a deep learning model to the current signals, vibration signals, and flux signals of different types of faults on a t-SNE dimensionality-reduction chart. By observing the distribution of the output feature data in low-dimensional space, we can evaluate the stability and consistency of the deep learning model’s predictive ability on different datasets. If the model data corresponding to different datasets cluster together in the space mapped by t-SNE, and the inter-class distance between different fault samples is large, it indicates that the model has good robustness. On the other hand, if the data corresponding to different datasets are scattered in the space mapped by t-SNE, and the inter-class distance between faults is relatively small, along with an overlap between different fault samples, it indicates that the model’s fault recognition performance is relatively poor.

### 4.3. Hypothesis Test

The data-based cross-validation approach is primarily utilized to select the most appropriate deep learning model for a given task. Its objective is to compute the given evaluation metrics by independently testing a subset of the input data. This process aims to reduce the dependence of external variables, as in random initialization methods. However, how can we determine which model is better for a given task? The statistical hypothesis test provides a crucial criterion for comparing the performance of different learners. Based on the hypothesis test results, we can infer whether the generalization performance of learner A is statistically better than that of learner B and how certain this conclusion is if learner A is observed to be better than B on the testing set. We used the Friedman test [61] based on algorithmic ranking and the post-hoc Nemenyi test [62].

If we assume that *k* learners are compared on *N* datasets and let *r*_i_ denote the average ranking value of the *i*-th learner, then, *r*_i_ obeys a normal distribution with a mean and variance of (*k* + 1)/2 and (*k*^2^ − 1)/12, respectively. The variable $T_{F}$ is obtained as follows:

(9) $$\begin{array}{l} T_{F}=\frac{\left(N-1\right)T_{\chi^{2}}}{N\left(k-1\right)-T_{\chi^{2}}}\\ \mathrm{where}\;T_{\chi^{2}}=\frac{12N}{k\left(k+1\right)}\left(\sum_{i=1}^{k}r_{i}^{2}-\frac{k{\left(k+1\right)}^{2}}{4}\right) \end{array}.$$

We can see that the variable $T_{F}$ obeys the *F*-distribution with degrees of freedom *k* − 1 and (*k* − l)(*N* − 1), respectively, while $T_{\chi^{2}}$ follows the distribution of $\chi^{2}$ with degrees of freedom *k* − 1. The confidence for our test is set to 90%, so, for *p* < 0.1, there is evidence to reject the null hypothesis H_0_ and accept the alternative hypothesis, H_1_. For *p* ≥ 0.1, there is evidence to accept the null hypothesis H_0_. The hypothesis for our test is as follows:

**H_0_.**  *All learners have the same performance.*

**H_1_.**  *The performance of the algorithms is significantly different.*

Some frequently used critical values of $T_{F}$ in the Friedman test are given in Table 5.

When the null hypothesis H_0_ is rejected in the Friedman test, it indicates that the learners have significantly different performances. In such cases, we use the post-hoc Nimenyi test to further distinguish the performance ranking of each learner. The Nemenyi test calculates the critical domain for the difference in the mean value of the ranking of *CD* based on the following equation:

(10) $$CD=q_{\alpha }\sqrt{\frac{k\left(k+1\right)}{6N}}$$

If the difference between the average rank values of the two learners exceeds the critical value of CD, the hypothesis that the two learners perform equally is rejected with the corresponding confidence level. Table 6 gives the frequently used values of $q_{\alpha }$ for *p* = 0.1.

## 5. Implementation Details

### 5.1. Cross-Validation Experimental Setup

**Figure 11:**
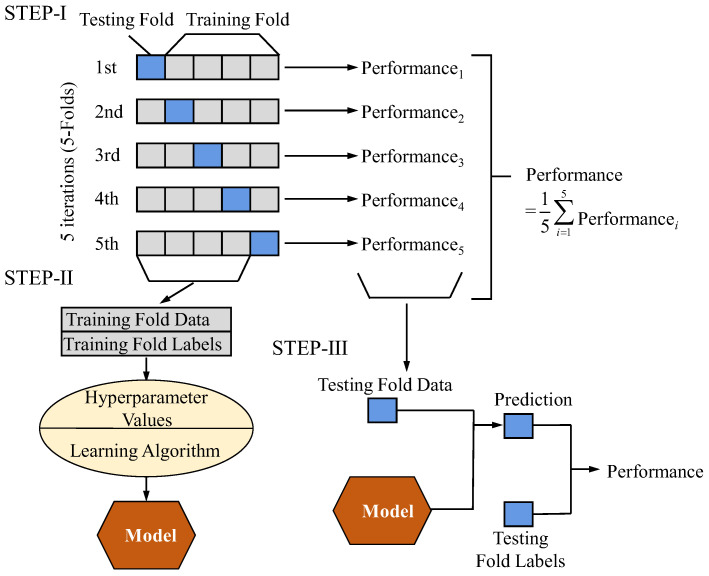
Schematic diagram of the procedure of the five-fold cross-validation experiment.

Two datasets, SENSORC and SENSORA_SENSORV, were utilized to train and evaluate machine learning models. The SENSORC dataset, obtained from three-phase current sensors, was primarily used for training and evaluating the performance of our deep learning models. The SENSORA_SENSORV dataset, which includes data from vibration and axial magnetic flux sensors, was employed for comparative analysis, allowing us to assess the models’ robustness across different types of sensor data. We adopted a five-fold cross-validation approach to evaluate the models’ performance and perform hyperparameter optimization. Specifically, the dataset was divided into two parts, namely, the training set and the testing set. The training set was used for constructing the model and hyperparameter optimization, while the testing set was used to assess the model’s generalization capability. The complete experimental scheme of the five-fold cross-validation experiment is presented in Figure 11.

**Figure 12:**
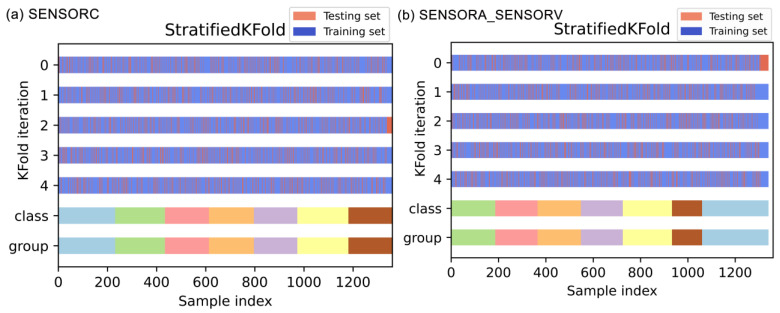
Random data assignment for the five-fold cross-validation experiment. (**a**) SENSORC dataset; (**b**) SENSORA_SENSORV dataset.

Moreover, as indicated in Table 1, the multi-classification task showed an imbalance in the distribution of target fault classes. To mitigate potential bias in evaluating the model generalization arising from this imbalance, we employed stratified sampling by implementing stratified five-fold cross-validation, ensuring that the relative frequencies of each fault class were approximately maintained in each training and testing fold. Specifically, each test fold contained one-fifth of the samples, which were selected randomly without replacement while maintaining the class proportions of the full dataset. The sample extraction process is illustrated in Figure 12. This ensured that the distribution of fault types in each fold mirrored that of the entire dataset, thereby reducing potential evaluation bias in the assessment of the models’ generalization capability.

Furthermore, to address the considerable variation in the scale of sample feature values, often arising from differences in units of measurement, this study employed a normalization strategy to bring all input features to a common scale. Variability in feature magnitudes can hinder the effective training of deep learning models, especially those that are sensitive to the scale of features. In this study, we utilized the Z-normalization, which is defined as follows:

(11) $$Z_{norm}=\frac{x-\mu }{\sigma }$$

where *x* is the original feature value, *μ* is the mean, and *σ* is the standard deviation, all of which are calculated from the training data. To prevent data leakage and ensure a fair evaluation of a model’s generalization, the mean and standard deviation used to normalize the test set must be derived solely from the training set. This approach standardizes each feature to have a zero mean and unit variance, thus enhancing the model’s ability to learn effectively across varying feature dimensions.

### 5.2. Model Training Details

**Figure 13:**
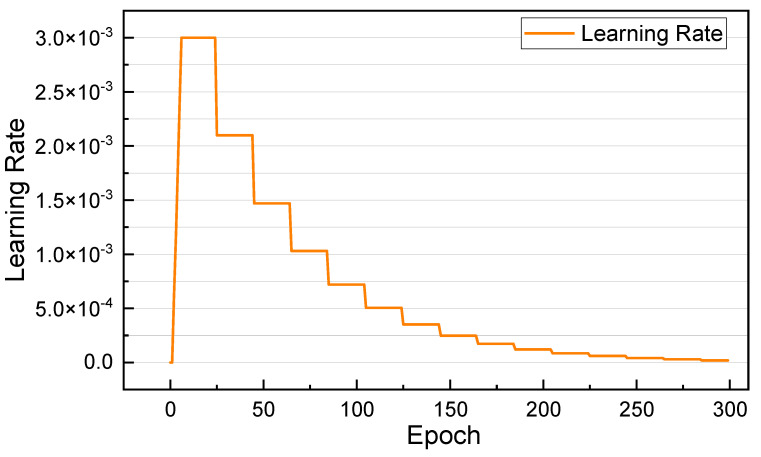
Learning rate adjustment schedule for training model.

AdaBound [63] is an adaptive learning rate-optimization algorithm that introduces upper and lower bound limits while adaptively adjusting the learning rate, thereby effectively controlling the update range of the gradient and avoiding the problem of an unstable gradient. For the optimization algorithm for parameter learning, we employed the AdaBound algorithm with a batch size of 16 and used the weight initialization method proposed by He, Zhang, Ren, & Sun (2015) [64] to initialize the network’s weights and train all networks from scratch. We used a stepwise decay schedule for the learning rate, starting with an initial learning rate of 3 × 10^−3^ and then decaying it by 0.7 every 25 epochs. Moreover, in the initial phase of model training, the gradient is often quite large due to the randomly initialized parameter settings, which can destabilize the training process if the initial learning rate is set too large. Therefore, we gradually warmed up the learning rate in the first five epochs of the training schedule, as shown in Figure 13. The comparison results of the effects of different optimization algorithms, batch sizes, and learning rates on the models’ performance are detailed in Appendix A to illustrate our choice of the above hyperparameters.

## 6. Methods for Comparison and Analysis

### 6.1. Confusion Matrix Results for Fault Diagnosis

**Figure 14:**
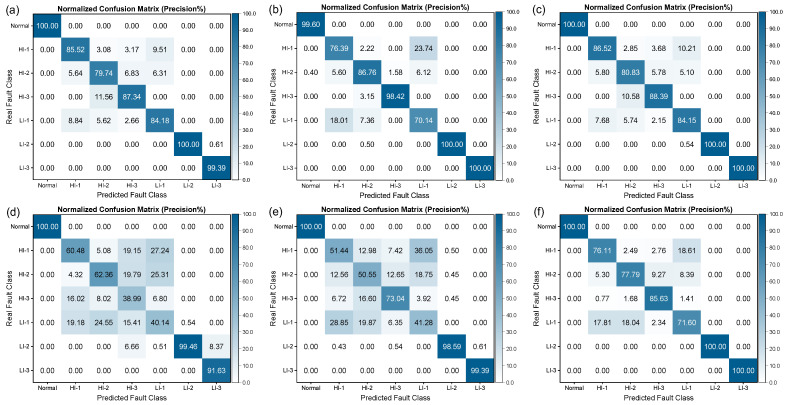
Column-normalized confusion matrix (Precision%) of different algorithms for short-circuit fault identification on SENSORC dataset. (**a**) SVM (LINEAR) algorithm; (**b**) SVM (RBF) algorithm; (**c**) SVM (POLY) algorithm; (**d**) Bayes (Gaussian) algorithm; (**e**) KNN algorithm; (**f**) Random Forest algorithm.

Figure 14 illustrates the confusion matrix of different machine learning algorithms for fault diagnosis, in which each row corresponds to the true label and each column represents the predicted label. The precision percentage is calculated by normalizing each column, indicating the proportion of true labels for that class among all samples predicted as being in that class. The values on the diagonal of the matrix denote the precision of the algorithm in recognizing that class of fault. The results indicate that Normal, LI-2, and LI-3 are the easiest fault types to correctly recognize, with the recognition precisions of all algorithms being above 90% for these. Conversely, HI-1, HI-2, HI-3, and LI-1 faults are relatively more challenging to recognize.

**Figure 15:**
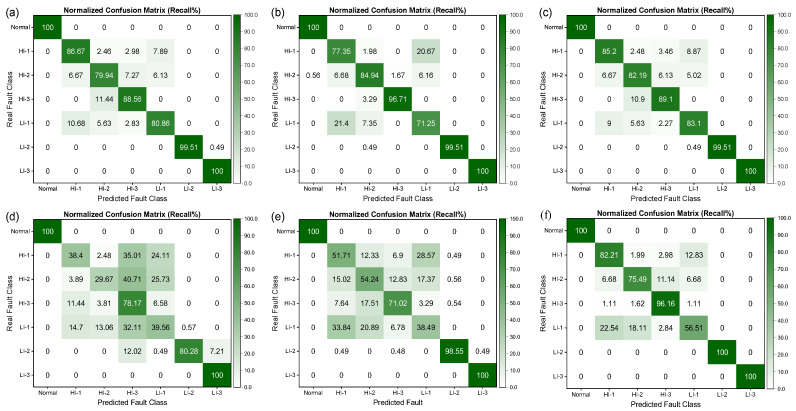
Row-normalized confusion matrix (Recall%) of different machine learning algorithms for short-circuit fault identification on SENSORC dataset. (**a**) SVM (LINEAR) algorithm; (**b**) SVM (RBF) algorithm; (**c**) SVM (POLY) algorithm; (**d**) Bayes (Gaussian) algorithm; (**e**) KNN algorithm; (**f**) Random Forest algorithm.

Among the well-performing SVM algorithms, the SVM-RBF algorithm had the lowest recognition precision of 70.14% for LI-1, and 23.74% of true HI-1 faults were misdiagnosed as LI-1 faults. Moreover, the Bayesian and KNN algorithms had lower recognition precisions of only 40.14% and 41.28%, respectively, for LI-1 faults and were more prone to confusion with HI-1 faults. To comprehenWe have modifiedsively evaluate the difficulty of recognizing different fault types and the performance of different algorithms, a row-normalized confusion matrix is presented in Figure 15, where the diagonal represents the recall rate of the algorithm for recognizing that class of fault.

**Figure 16:**
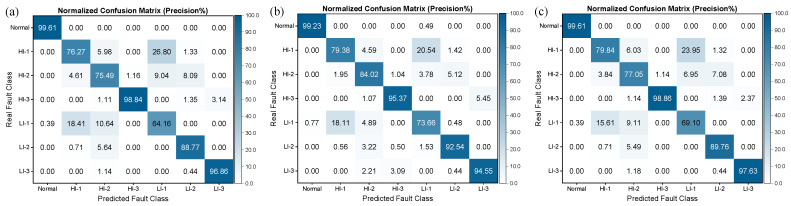

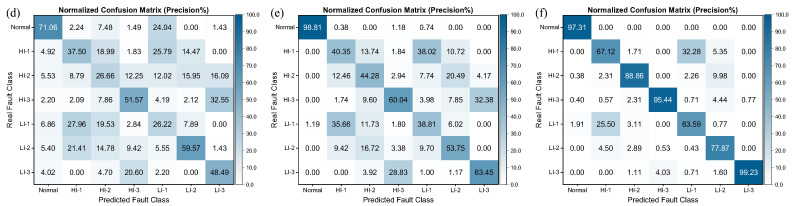
Column-normalized confusion matrix (Precision%) of different machine learning algorithms for short-circuit fault identification on SENSORA_SENSORV dataset. (**a**) SVM (LINEAR) algorithm; (**b**) SVM (RBF) algorithm; (**c**) SVM (POLY) algorithm; (**d**) Bayes (Gaussian) algorithm; (**e**) KNN algorithm; (**f**) Random Forest algorithm.

**Figure 17:**
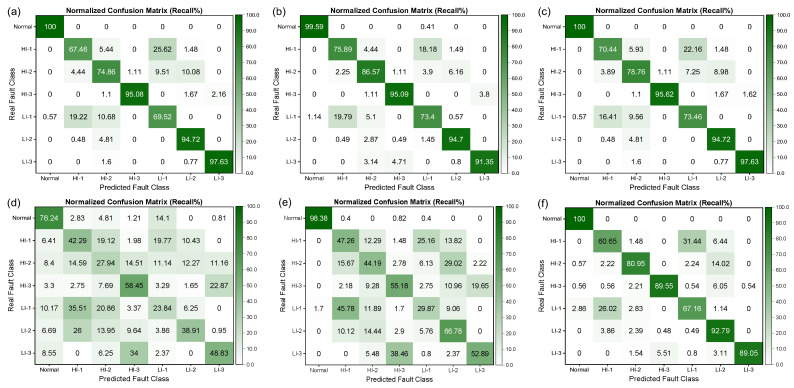
Row-normalized confusion matrix (Recall%) of different machine learning algorithms for short-circuit fault identification on SENSORA_SENSORV dataset. (**a**) SVM (LINEAR) algorithm; (**b**) SVM (RBF) algorithm; (**c**) SVM (POLY) algorithm; (**d**) Bayes (Gaussian) algorithm; (**e**) KNN algorithm; (**f**) Random Forest algorithm.

From Figure 15, it can be observed that the fault recognition performance of the three SVMs was relatively better, with recognition precision and recall rates of over 70% for all fault categories. However, the Bayesian and KNN algorithms performed poorly in distinguishing between LI-1 and HI-1 faults, which are more easily confused, resulting in lower recognition precision and recall rates. It is noteworthy that, as demonstrated in Figure 16 and Figure 17, the performance of all algorithms on the SENSORA_SENSORV dataset was decreased to some extent. The recognition difficulty for HI-1, HI-2, and LI-1 faults is relatively high, and the algorithms were more likely to confuse them, resulting in lower recognition precision and recall rates. In general, the conventional machine learning algorithm can identify the normal state and fault states of the generator, but its performance in diagnosing and identifying short-circuit fault categories is unsatisfactory. Particularly, the diagnosis of four fault categories (HI-1, LI-1, HI-2, and HI-3) was poorly differentiated, which can easily lead to misjudgment and confusion.

**Figure 18:**
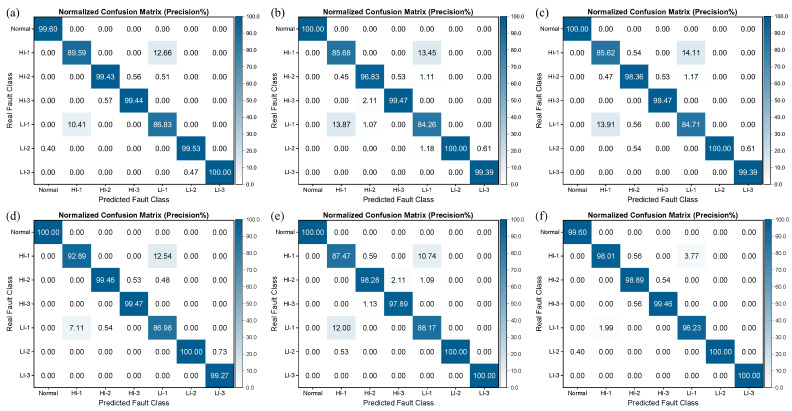
Column-normalized confusion matrix (Precision%) of different deep learning models for short-circuit fault identification on the SENSORC dataset. (**a**) ResNet-31; (**b**) ResNeXt-22; (**c**) DenseNet-37; (**d**) VggNet-18; (**e**) Inception-ResNet-59; (**f**) FCNet-5.

Figure 18 shows the confusion matrices obtained for different deep learning models for detecting generator short circuit faults using the SENSORC dataset. The results demonstrate that deep learning models achieve quantitatively higher precision compared to machine learning algorithms, successfully achieving a precision exceeding 84% for all types of fault categories. Moreover, these models exhibit an excellent capacity to recognize the Normal category, obtaining a precision that was almost 100%, underscoring their high capacity to identify the normal state of generators.

However, in terms of the results for the classification of fault types, the precision of different models varies across different categories. Specifically, for the HI-1 and LI-1 fault categories, the different models exhibited significant differences in precision. Among these models, the FCNet-5 model achieved an outstanding performance with a precision exceeding 96%, whereas the ResNet-31, ResNeXt-22, DenseNet-37, and Inception-ResNet-59 models demonstrated a precision below 90%. Additionally, the VGG-18 achieved a precision of over 90% for HI-1 but only 88.17% for LI-1.

**Figure 19:**
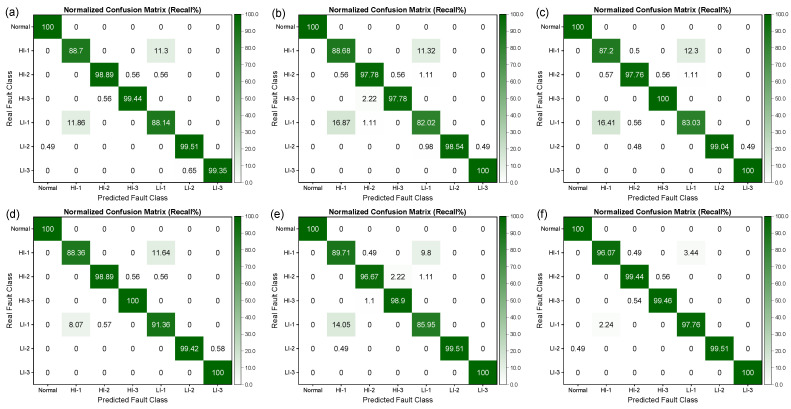
Row-normalized confusion matrix (Recall%) of different deep learning models for short-circuit fault identification on the SENSORC dataset. (**a**) ResNet-31; (**b**) ResNeXt-22; (**c**) DenseNet-37; (**d**) VggNet-18; (**e**) Inception-ResNet-59; (**f**) FCNet-5.

Furthermore, Figure 19 illustrates that all deep learning models manifest high recall rates for various fault categories. Notably, the five generator fault states of Normal, HI-2, HI-3, LI-2, and LI-3 yielded recall rates above 90%. Nonetheless, the recall rates for the HI-1 and LI-1 fault categories were relatively lower, albeit still above 82%. The observed variation in recall rates across different models further emphasizes the challenging nature of recognizing and diagnosing HI-1 and LI-1 faults, thereby implying the need for more robust deep learning models rather than machine learning models. Deep learning models significantly outperform traditional machine learning algorithms in terms of fault diagnosis on the SENSORC dataset. However, the precision and recall rates of different models exhibit variations across distinct fault categories, especially in the case of the HI-1 and LI-1 fault types.

**Figure 20:**
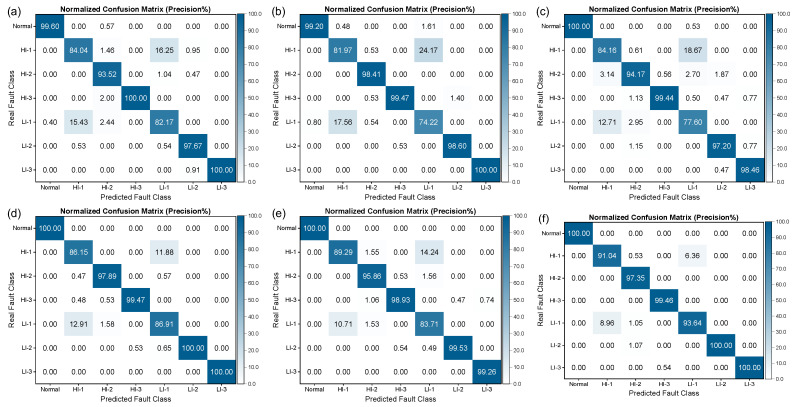
Column-normalized confusion matrix (Precision%) of different deep learning models for short-circuit fault identification on SENSORA_SENSORV dataset. (**a**) ResNet-31; (**b**) ResNeXt-22; (**c**) DenseNet-37; (**d**) VggNet-18; (**e**) Inception-ResNet-59; (**f**) FCNet-5.

**Figure 21:**
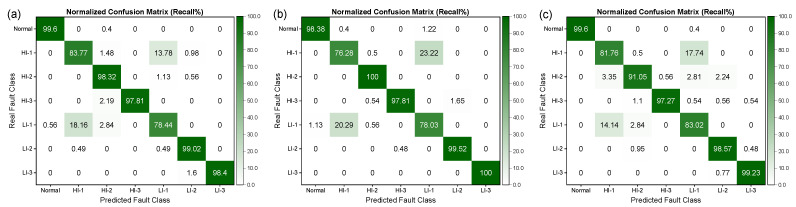

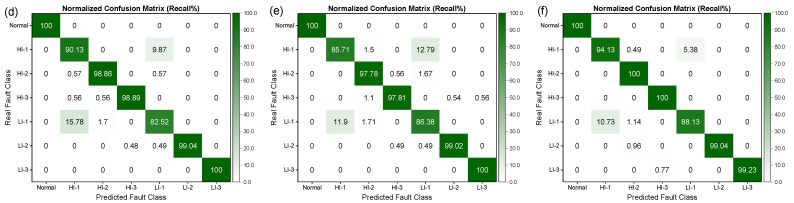
Row-normalized confusion matrix (Recall%) of different deep learning models for short-circuit fault identification on SENSORA_SENSORV dataset. (**a**) ResNet-31; (**b**) ResNeXt-22; (**c**) DenseNet-37; (**d**) VggNet-18; (**e**) Inception-ResNet-59; (**f**) FCNet-5.

Figure 20 and Figure 21 illustrate the confusion matrices obtained for different deep learning models for identifying short-circuit faults in generators using the SENSORA_SENSORV dataset. It is apparent that the precision and recall rates of the models exhibit varying degrees of decline, particularly for the HI-1 and LI-1 faults, where the precision and recall rates of some models have fallen below 80%. These findings suggest that utilizing different sensors to diagnose generator faults entails varying levels of complexity. As a result, even though different deep learning models perform differently in identifying short-circuit faults in generators, they all demonstrate an improved performance compared to machine learning algorithms. Hence, selecting an appropriate model that suits specific problems and dataset characteristics is imperative. Additionally, a model’s computational complexity, performance, robustness, and generalizability should be considered in evaluating its effectiveness and practicality in real-world applications.

### 6.2. Average Accuracy (AP%) Results for Different Fault Types

**Figure 22:**
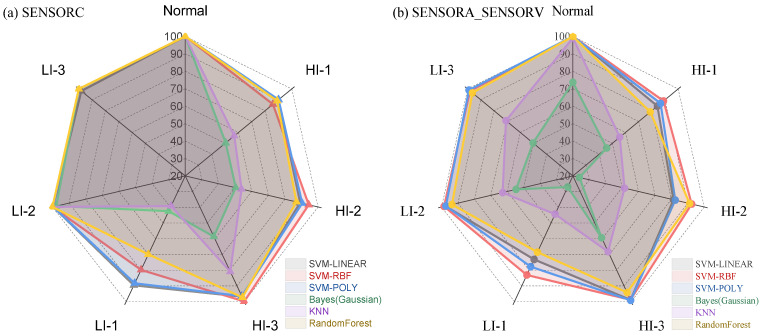
A comparison of the average accuracy (AP%) of various machine learning algorithms in identifying different short-circuit fault types of WT generators. (**a**) Comparison on SENSORC dataset; (**b**) Comparison on SENSORA_SENSORV dataset.

Table 7 and Figure 22 present a comparison of the average accuracy (AP%) of various machine learning algorithms in identifying different short-circuit fault types of WT generators. Among the algorithms that were considered, SVMs (LINEAR, RBF, and POLY kernel) and random forest algorithms exhibited a relatively better performance on the SENSORC dataset. SVM-LINEAR shows an AP% of over 89% in all categories, while SVM-RBF performs best in the LI-2 and LI-3 categories, with an AP% of 100%. However, the AP% of SVM-RBF for LI-1 was slightly lower at 79.78%. SVM-POLY performs similarly to SVM-LINEAR, with an AP% above 88% in all categories. On the other hand, except for the AP% of 69.92% for LI-1, the random forest algorithm exhibited an AP% of over 86% for all fault types. In contrast, the average accuracy of the Bayes (Gaussian) algorithm and KNN algorithm was relatively low. The performance of the Bayes (Gaussian) algorithm was highly unstable, with an AP% of only about 50% for the HI-1 and HI-2 fault types and a relatively low AP% for the LI-1 fault type. The performance of the KNN algorithm was slightly better than that of the Bayes (Gaussian) algorithm, but its AP% for the HI-1, HI-2, and LI-1 fault types was also very low, all below 60%, and its AP% for the LI-1 fault type was only 39.17%.

On the SENSORA_SENSORV dataset, the performance of all algorithms declined, and SVM-RBF performed the best, with an AP% of over 83% for all categories. SVM-POLY and SVM-LINEAR performed well in most categories but exhibited poor performance in the LI-1 category, with an AP% below 78%. The random forest also performed poorly in the HI-1 and LI-1 categories, with an AP% below 79%. The performance of the Bayes (Gaussian) and KNN algorithms was relatively poor compared with the other algorithms, with an AP% below 71% for all faults, and the Bayes (Gaussian) algorithm performed the worst in the HI-2 and LI-1 categories, with an AP% below 28%.

In addition, as shown in Figure 22, machine learning algorithms exhibit varying degrees of difficulty in identifying different types of faults. The HI-3 and LI-3 faults were relatively easy to identify, and most machine learning algorithms could achieve good identification to some extent. However, identifying the HI-1 and LI-1 faults was more challenging, particularly LI-1, where some algorithms exhibited an AP% of less than 30%. Furthermore, Table 7 shows the identification performance of different sensor datasets. For the SENSORC dataset, the identification performance of different algorithms was relatively stable for all fault types. In contrast, for the SENSORA_SENSORV dataset, the identification results varied significantly across algorithms, and the identification performance was relatively poor.

**Figure 23:**
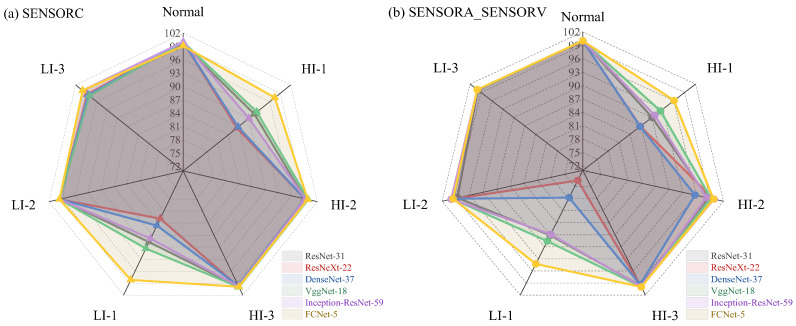
A comparison of the average accuracy (AP%) of various deep learning models in identifying different short-circuit fault types of WT generators. (**a**) Comparison on the SENSORC dataset; (**b**) Comparison on the SENSORA_SENSORV dataset.

Table 8 and Figure 23 display the AP scores of different deep learning models for the seven fault categories. On the SENSORC dataset, all models demonstrated AP scores exceeding 99% for the Normal class and over 98% for the LI-3 fault class. VggNet-18 and FCNet-5 excelled across all fault types, with VggNet-18 achieving an AP% above 90% for all fault types and FCNet-5 scoring above 97% for all fault types, including a perfect 100% for the LI-3 fault class. However, ResNeXt-22 and DenseNet-37 exhibited a comparatively poor performance on the HI-1 and LI-1 fault classes, recording an AP% below 87% for HI-1 and below 85% for LI-1. In addition, on the SENSORA_SENSORV dataset, all models displayed varying degrees of decline in their performance for most fault types, particularly for the LI-1 fault class. ResNet-22 attained an AP score of 73.52%, while DenseNet-37 attained an AP score of 77.76%. The performance degradation for the Normal, HI-3, LI-2, and LI-3 fault types was slightly lower, with all models recording AP values above 98% for these four fault types.

The AP% scores for different fault categories in Figure 23 indicate the efficacy of the deep learning algorithms in identifying stator winding short-circuit faults in WT generators. The deep learning models demonstrated remarkable precision in fault detection, with all models achieving AP% scores above 99% for the “Normal” category, indicating their ability to effectively differentiate between normal operating conditions and fault states. Regarding different fault types, all models achieved an AP% above 98% for the HI-3, LI-2, and LI-3 categories, indicating their ability to accurately detect these three types of faults. This ability may be due to the distinct characteristics exhibited by these faults in terms of their machine vibration and current signals, which made them easier for the models to identify. However, we observed relatively lower average precisions for the “HI-1” and “LI-1” fault categories, possibly due to the less distinctive nature of these faults, which may require more data for accurate identification. Finally, it should be noted that the generalization performance of these models also depends on the quantity and quality of their training data. Therefore, more comprehensive and in-depth evaluations of these models in practical applications are necessary to determine their performance and applicability.

### 6.3. Comprehensive Performance Evaluation

**Figure 24:**
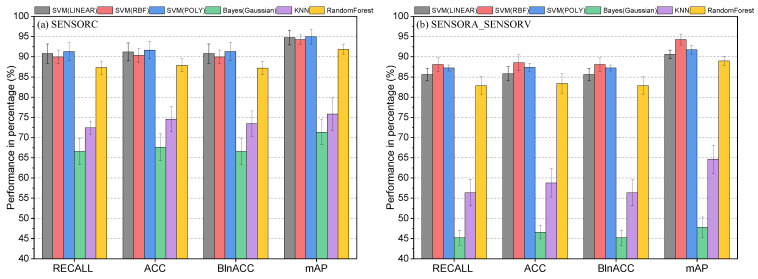
Performance comparison of different machine learning models. (**a**) Comparison on the SENSORC dataset; (**b**) Comparison on the SENSORA_SENSORV dataset.

As shown in Table 9 and Figure 24, four evaluation metrics were used to assess the performance of various algorithms: macro average recall (Recall%), accuracy (ACC%), balance accuracy (BlnACC%), and mean average precision (mAP%). Regarding the SENSORC dataset, the results indicate that SVM-LINEAR, SVM-RBF, and SVM-POLY achieved relatively good performance, with mAP% exceeding 90%. Moreover, the ACC% and BlnACC% were around 90%, with SVM-POLY achieving the highest ACC% and BlnACC% at 95.00% and 91.30%, respectively. These algorithms also demonstrated high Recall% scores of 90.79%, 89.97%, and 91.3%. Among the three algorithms, SVC-POLY achieved the top performance with a Recall% of 91.3%, an ACC% of 95.00%, a BlnACC% of 91.30%, and an mAP% of 91.66%, respectively. RandomForest also achieved good results, but its results were slightly lower than those of the SVM algorithms, as it obtained an mAP% of 87.9%. However, the Bayesian and KNN algorithms performed relatively poorly on the SENSORC dataset, with a Recall% of 66.58% and 73.43%, an ACC% of 71.35% and 75.82%, a BlnACC% of 66.58% and 72.42%, and an mAP% of 67.62% and 74.55%, respectively.

Moving on to the SENSORA_SENSORV dataset, the SVM-LINEAR, SVM-RBF, SVM-POLY, and RandomForest models still performed relatively well, with recall rates ranging from 82.8% to 88.1%, accuracy rates ranging from 83.4% to 88.6%, balance accuracy rates ranging from 82.9% to 88.1%, and mAP% rates ranging from 88.9% to 94.3%. Among these models, the SVM-RBF algorithm achieved the best performance with an mAP% of 94.27%. However, compared to their results for the SENSORC dataset, the performance of all of these algorithms decreased. In contrast, the KNN and Gaussian Bayes algorithms performed the worst in this fault diagnosis task, with mAP% scores of 64.63% and 47.82%, respectively.

**Figure 25:**
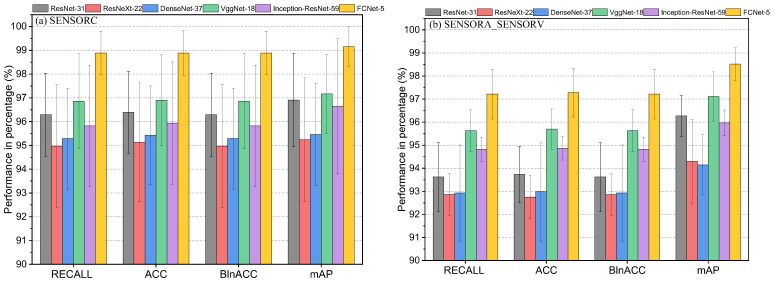
Performance comparison of different deep learning models. (**a**) Comparison on the SENSORC dataset; (**b**) Comparison on the SENSORA_SENSORV dataset.

In Table 10 and Figure 25, it is evident that deep learning algorithms outperform traditional machine learning algorithms in detecting short-circuit faults in motor stators for WTs. On the SENSORC dataset, all deep learning models demonstrated outstanding performance with minimal performance differences. Their Recall% and mAP% were both above 95%. Among them, ResNet-31, VggNet-18, and FCNet-5 performed the best in fault recognition, with FCNet-5 achieving state-of-the-art performance in all metrics, reaching as high as 98.89%, 98.89%, 98.89%, and 99.15%, respectively. ResNet-31 and VggNet-18 also exhibited good performance, with accuracy, recall, and mAP scores all above 96%. However, the performance of the ResNeXt-22, DenseNet-37, and Inception-ResNet-59 models on this dataset was relatively poor. Nevertheless, their recognition accuracy, recall rate, and mAP still remained above 95%, indicating that their fault recognition capabilities are also strong.

Moreover, all deep learning models demonstrated a performance decline on the SENSORA_SENSORV dataset. Among them, the ResNet-31, VggNet-18, and FCNet-5 models still outperformed the other models, with mAP% scores above 96%. Further, FCNet-5 also achieved the highest level in all metrics, reaching 97.22%, 97.28%, 97.22%, and 98.52%, respectively. The performance of the ResNeXt-22, DenseNet-37, and Inception-ResNet-59 models on this dataset was relatively poor, but their recognition accuracy, recall rate, and mAP were still above 92%.

**Figure 26:**
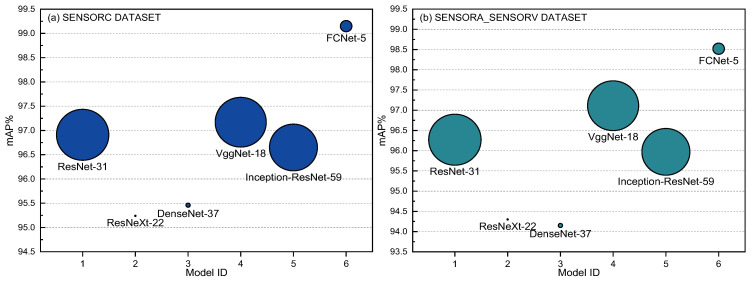
Performance comparison of different deep learning models. Circle’s area: the parameter magnitude. (**a**) Comparison on the SENSORC dataset; (**b**) Comparison on the SENSORA_SENSORV dataset.

The deep learning models exhibited a high fault detection performance, especially FCNet-5, which displayed exceptional performance on both datasets, with its mAP% exceeding 98%. Nevertheless, the other deep learning models also showed excellent performance, with mAP% scores above 94%. These findings indicate that deep learning algorithms are capable of providing more accurate and reliable fault diagnosis results, as these methods can capture more abstract features and automatically extract information that is beneficial for diagnosis. Notably, the complexity of deep learning models varies significantly, as demonstrated by the substantial differences in FLOPs and Params shown in Table 10 and Figure 26. For instance, ResNeXt-22 and DenseNet-37 had relatively small FLOPs and Params, but their performance was comparatively low. Thus, when choosing a model, a trade-off between the model performance and computational cost is necessary.

### 6.4. Deep Learning Model Robustness Discussion

Robustness is a crucial aspect of model selection, referring to a model’s ability to handle changes and perturbations in input data. Selecting a model with good robustness can result in more stable and reliable performance when it is presented with different types of data. Deep learning models with improved robustness can adapt better to variations, noise, and interference in the input data, resulting in enhanced accuracy and reliability in practical applications. Moreover, deep learning models exhibiting good robustness and can also withstand adversarial attacks, ensuring security and reliability.

**Figure 27:**
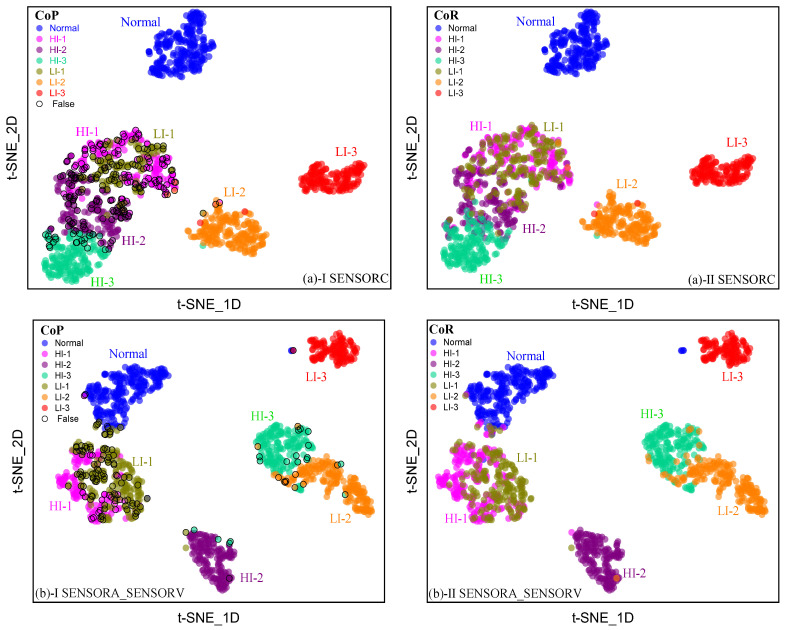
Embedding visualization via t-SNE for the output of ResNet-6. CoP: colors on the predicted fault type; CoR: colors on the real fault type. The black coils highlight the predicted fault type as wrong. (**a**)-I CoP for the SENSORC dataset; (**a**)-II CoR for the SENSORC dataset; (**b**)-I CoP for the SENSORA_SENSORV dataset; (**b**)-II CoR for the SENSORA_SENSORV dataset.

Figure 27 illustrates the outputs of ResNet-6 on the SENSORC and SENSORA_SENSORC datasets, which were then visualized in two-dimensional space through t-SNE mapping. The results reveal that the shallow residual network achieves high accuracy in recognizing the Normal, LI-2, and LI-3 fault states of the generator stator on the SENSORC dataset while maintaining a low misclassification rate. However, the detection robustness of four fault categories, namely HI-I, LI-1, HI-2, and HI-3, is notably poor, making it challenging to achieve precise identification. This is attributed to the relatively small fault class spacing for the network output and the cross-mixing between different fault samples, which led to significant confusion between HI-I and LI-1 faults. Moreover, HI-3 and LI-2 faults are comparatively easy to misinterpret.

In addition, we found that the class spacing between the HI-1 and LI-1 faults for the network outputs obtained using the SENSORA_SENSORC dataset was small. This resulted in difficulty distinguishing between the two types of faults. Additionally, there was a problem of small inter-class distance in the network output for the HI-3 and LI-2 faults, making it easy to confuse them with each other. Therefore, through low-dimensional space visualization of the network output, we could study the performance of ResNet-6 in recognizing the four types of fault categories—HI-I, LI-1, LI-2, and HI-3. We observed poor robustness in ResNet-6 for these categories, and found that it was prone to misjudgment. Consequently, it is necessary to develop networks with better performance and robustness for the diagnosis and recognition of these four types of faults.

**Figure 28:**
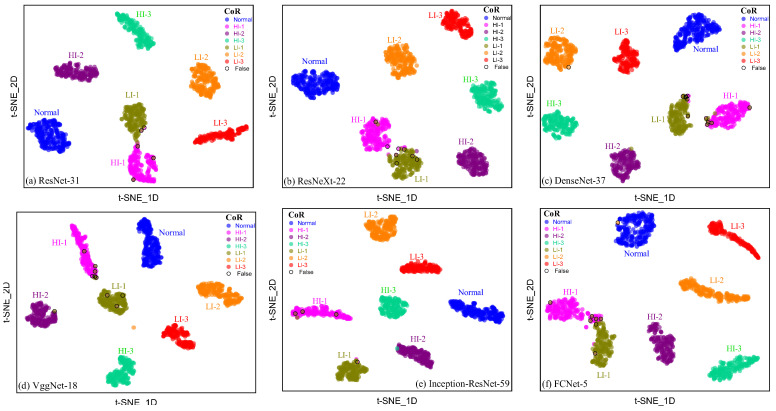
Embedding visualization via t-SNE for the outputs of deep learning models on the SENSORC dataset. CoP: colors on the predicted fault type; CoR: colors on the real fault type. The black coils highlight the predicted fault type as wrong. (**a**) ResNet-31; (**b**) ResNeXt-22; (**c**) DenseNet-37; (**d**) VggNet-18; (**e**) Inception-ResNet-59; (**f**) FCNet-5.

**Figure 29:**
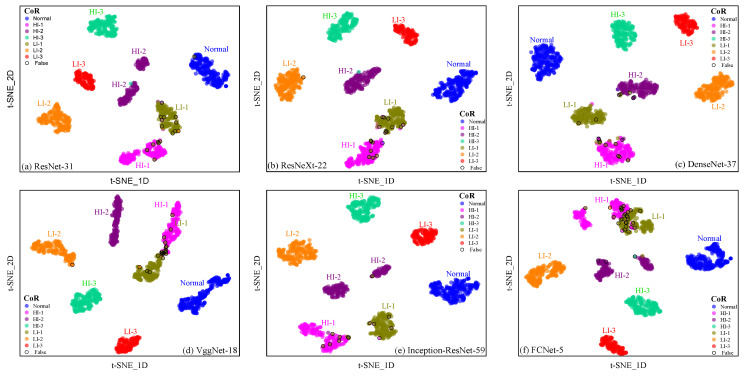
Embedding visualization via t-SNE for the outputs of deep learning models on the SENSORA_ SENSORV dataset. CoR: colors on the real fault type. The black coils highlight the predicted fault type as wrong. (**a**) ResNet-31; (**b**) ResNeXt-22; (**c**) DenseNet-37; (**d**) VggNet-18; (**e**) Inception-ResNet-59; (**f**) FCNet-5.

Figure 28 and Figure 29 compare the visualization results of output features from deep networks with various architectures in a two-dimensional space using the t-SNE method. It was observed that deep learning models demonstrate robustness in detecting short-circuit faults; specifically, they exhibit larger inter-class distances between different types of faults and good intra-class clustering of faults of the same type. Notably, for the Normal, HI-2, HI-3, LI-2, and LI-3 fault states, there was a relatively large inter-class distance between the network output features, which reduced the misclassification rates and improved the models’ ability to resist input interference. However, for the two fault categories of HI-1 and LI-1, the inter-class distance between network output features was relatively small, particularly for the FCNet-5 network, which misdiagnosed some samples. Nonetheless, the model could still distinguish between these two fault types, as there was minimal overlap between fault samples from different categories and good intra-class clustering of faults of the same type. Therefore, all six deep learning models designed by us can perform well in diagnosing stator short-circuit faults in generators, demonstrating high performance with mAP% values exceeding 94%. These models exhibit strong robustness. However, special attention should be paid to the recognition effect of the HI-1 and LI-1 fault types when selecting the network model.

### 6.5. Deep Learning Model Selection

We employed the mAP% metric from Table 10 to evaluate and rank the performance of six distinct deep learning models. The average ranking of each model’s performance was then calculated by conducting five-fold cross-validation experiments. As shown in Table 11, we evaluated the performance rankings and averages of the six deep learning models in evaluating the SENSORC dataset during the course of these experiments.

**Figure 30:**
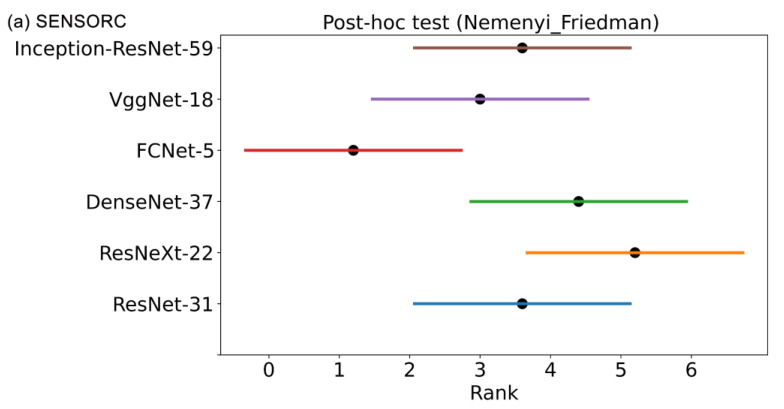

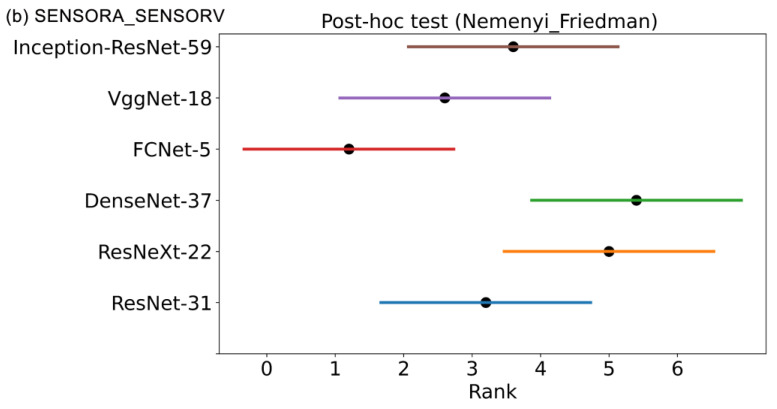
Post-hoc test (Nemnyi Friedman) for model performance ranking. (**a**) Ranking on the SENSORC dataset; (**b**) Ranking on the SENSORA_SENSORV dataset.

According to the significance level of *p* = 0.1 listed in Table 5, the critical value of $T_{F}$ was found to be 2.158 when the number of cross-validation experiments *N* was 5 and the number of deep learning models *k* was 6. Using Equation (10) with ranked data from Table 11, we calculated the values of $T_{\chi^{2}}$ and $T_{F}$ to be 13.23 and 4.495, respectively. Since the value of $T_{F}$ exceeds the critical value of 2.158, we could reject the null hypothesis H_0_ in favor of the alternative hypothesis H_1_. This result indicates that there exists statistically significant differences in the fault identification performances of the six deep learning models under consideration. To further differentiate the performance ranking differences among the models, we conducted the post-hoc Nimenyi test. By applying Equation (11) along with the corresponding values of $q_{\alpha }$ in Table 6, we calculated the critical value of CD as 3.063 and plotted the performance ranking of the six deep learning models, as shown in Figure 30. Each model is represented by a line segment range which corresponds to its upper and lower limits of performance. If two models’ performance ranking ranges overlap, their performance difference is not considered statistically significant. Additionally, we have provided the performance ranking of the models based on their evaluation of the SENSORA_SENSORV dataset, which is shown in Figure 30.

Through a five-fold cross-validation experiment and statistical hypothesis testing of the models’ performance, it has been found that there are differences in the fault diagnosis performance of different deep learning models. Specifically, FCNet-5 specifically outperforms DenseNet-37 and ResNeXt-22. However, no apparent performance differences were found among the other deep learning models. Given the importance of effectively monitoring the health condition of WTs at field sites, it is crucial to ensure that the deployed sensors and models are reliable. Therefore, when selecting an appropriate deep learning model, it is important to consider a range of factors, such as the model’s robustness, computational complexity, parameter quantity, and performance. Making trade-offs between these dimensions will be necessary.

To further substantiate the effectiveness of our proposed deep learning-based fault diagnosis framework, we conducted a comparative analysis with existing methods reported in the literature. Table 12 provides a summary of the performance metrics for various methods that are applied to the diagnosis of ITSC faults in wind turbine generators.

This comparison clearly shows that our proposed FCNet-5 model not only achieves high accuracy but also outperforms other methods, particularly in robustness across different fault categories. The inclusion of this comparison table strengthens our manuscript by providing a direct benchmark against existing methodologies, demonstrating the superior performance of our approach.

## 7. Conclusions

This study presents a novel data-driven approach for the early detection of short-circuit faults in wind turbine stators, a critical issue that, if left undiagnosed, can cause significant equipment damage and reduce energy production. Our deep learning models, which integrate data from current, vibration, and axial magnetic flux sensors, effectively identify and classify seven distinct types of short-circuit faults, outperforming traditional machine learning algorithms through superior feature extraction. In particular, the FCNet-5 model achieved state-of-the-art mean average precision (mAP) scores of 99.15% and 98.52% on the SENSORC and SENSORA_SENSORV datasets, respectively, demonstrating the effectiveness of deep learning in fault diagnosis applications. Our findings underscore the particular effectiveness of three-phase current signals in monitoring faulty states of wind turbine generators, demonstrating that they provide more precise and reliable fault detection compared to vibration and electromagnetic signals. These findings are crucial for advancing the design and implementation of condition-monitoring systems in wind turbines. However, deep learning models require substantial computational resources and extensive training data, which may present challenges for real-time applications in resource-constrained environments. Additionally, distinguishing between HI-1 and LI-1 faults remains challenging due to their similar feature representations

In anticipation of future developments, it is essential to focus on developing lightweight model architectures that maintain high accuracy while reducing their computational demands, as well as enhancing the discriminatory ability of deep networks for closely related fault types. The proposed deep learning framework offers a versatile and effective solution for fault diagnosis in wind turbines and can be adapted to other scenarios. Integrating this approach with emerging technologies such as the internet of things (IoT) and edge computing could further enable the real-time, intelligent monitoring and maintenance of wind turbine operational states.

## Figures and Tables

**Table 1:** The components and fault categories of the short-circuit fault database.

Dataset Name	Sensor Setting	Total of Features	SCIG Condition	Count of Instances	Frequency of Instances
SENSORC	Three-phase current sensors	62	Normal state	248	18.29%
			HI-1 fault	203	14.97%
			HI-2 fault	179	13.20%
			HI-3 fault	183	13.50%
			LI-1 fault	177	13.05%
			LI-2 fault	208	15.34%
			LI-3 fault	158	11.65%
SENSORA_SENSORV	Tri-axial vibration and axial magnetic flux coil sensors	84	Normal state	248	18.72%
			HI-1 fault	203	15.32%
			HI-2 fault	179	13.51%
			HI-3 fault	183	13.81%
			LI-1 fault	177	13.36%
			LI-2 fault	208	15.70%
			LI-3 fault	127	9.58%

**Table 2:** Complexity comparison of different network models.

Deep Learning Model	FLOPs	Params
ResNet-31	5.894 M	244.967 k
ResNeXt-22	58.496 k	391.000 B
DenseNet-37	624.064 k	2.278 k
VggNet-18	8.532 M	232.807 k
Inception-ResNet-59	9.886 M	205.527 k
FCNet-5	209.920 k	13.367 k

**Table 3:** Confusion matrix for the fault multiclassification task.

Real Situation	Predicted Results						
	Normal (0)	HI-1 (1)	HI-2 (2)	HI-3 (3)	LI-1 (4)	LI-2 (5)	LI-3 (6)
Normal (0)	***C*_0,0_**	*C*_0,1_	*C*_0,2_	*C*_0,3_	*C*_0,4_	*C*_0,5_	*C*_0,6_
HI-1 (1)	*C*_1,0_	***C*_1,1_**	*C*_1,2_	*C*_1,3_	*C*_1,4_	*C*_1,5_	*C*_1,6_
HI-2 (2)	*C*_2,0_	*C*_2,1_	***C*_2,2_**	*C*_2,3_	*C*_2,4_	*C*_2,5_	*C*_2,6_
HI-3 (3)	*C*_3,0_	*C*_3,1_	*C*_3,2_	***C*_3,3_**	*C*_3,4_	*C*_3,5_	*C*_3,6_
LI-1 (4)	*C*_4,0_	*C*_4,1_	*C*_4,2_	*C*_4,3_	***C*_4,4_**	*C*_4,5_	*C*_4,6_
LI-2 (5)	*C*_5,0_	*C*_5,1_	*C*_5,2_	*C*_5,3_	*C*_5,4_	***C*_5,5_**	*C*_5,6_
LI-3 (6)	*C*_6,0_	*C*_6,1_	*C*_6,2_	*C*_6,3_	*C*_6,4_	*C*_6,5_	***C*_6,6_**

**Bold** indicates that the predicted results are consistent with the real.

**Table 4:** Confusion matrix for binary classification tasks.

Real Situations	Predicted Results	
	Predicted Positive	Predicted Negative
Actual Positive	True Positive (*TP*)	False Negative (*FN*)
Actual Negative	False Positive (*FP*)	True Negative (*TN*)

**Table 5:** Frequently used critical values of $T_{F}$ in Friedman test.

$T_{F}$ (*p =* 0.1)							
Number of Datasets *N*	Number of Learners *k*						
	2	3	4	5	6	7	8
4	5.538	3.463	2.813	2.480	2.273	2.130	2.023
5	4.545	3.113	2.606	2.333	2.158	2.035	1.943
8	3.589	2.726	2.365	2.157	2.019	1.919	1.843
10	3.360	2.624	2.299	2.108	1.980	1.886	1.814
15	3.102	2.503	2.219	2.048	1.931	1.845	1.779
20	2.990	2.448	2.182	2.020	1.909	1.826	1.762

**Table 6:** Frequently used values of $q_{\alpha }$ in Nemenyi test.

$q_{\alpha }$	Number of Learners *k*						
	2	3	4	5	6	7	8
0.05	1.960	2.344	2.569	2.728	2.850	2.949	3.031
0.1	1.645	2.052	2.291	2.459	2.589	2.693	2.780

**Table 7:** The average accuracy (AP%) of machine learning models for different types of short-circuit faults.

SENSORC DATASET							
Machine Learning Model	Normal	HI-1	HI-2	HI-3	LI-1	LI-2	LI-3
SVM-LINEAR	100 ± 0	89.96 ± 1.56	89.22 ± 5.83	96.87 ± 1.06	89.61 ± 4.94	99.59 ± 0.93	98.07 ± 4.32
SVM-RBF	100 ± 0	85.87 ± 3.99	94.36 ± 2.11	99.89 ± 0.08	79.78 ± 6.24	100 ± 0	100 ± 0
SVM-POLY	100 ± 0	90.13 ± 3.78	90.42 ± 5.48	96.53 ± 1.69	88.35 ± 6.99	99.59 ± 0.93	100 ± 0
Bayes (Gaussian)	100 ± 0	50.37 ± 8.82	50.4 ± 5.31	58.6 ± 10.02	42.45 ± 8.27	97.72 ± 2.81	99.92 ± 0.12
KNN	100 ± 0	56.83 ± 10.33	53.9 ± 8.95	80.93 ± 6.83	39.17 ± 6.71	99.9 ± 0.17	100 ± 0
RandomForest	100 ± 0	89.02 ± 5.45	86.91 ± 5.30	97.33 ± 1.55	69.92 ± 3.78	100 ± 0	100 ± 0
SENSORA_SENSORV DATASET							
Machine Learning Model	Normal	HI-1	HI-2	HI-3	LI-1	LI-2	LI-3
SVM-LINEAR	99.99 ± 0.02	84.23 ± 4.72	81.3 ± 7.40	98.95 ± 1.22	73.13 ± 4.10	97.7 ± 2.12	98.9 ± 2.21
SVM-RBF	100 ± 0	88.92 ± 4.30	92.38 ± 3.05	98.83 ± 0.74	83.04 ± 4.32	98.52 ± 0.83	98.17 ± 1.53
SVM-POLY	99.99 ± 0.02	86.66 ± 4.70	82.36 ± 6.61	99.17 ± 1.02	77.76 ± 3.30	97.41 ± 2.51	98.96 ± 2.24
Bayes (Gaussian)	73.71 ± 4.72	45.68 ± 9.34	23.97 ± 2.82	59.35 ± 3.35	27.07 ± 4.73	54.67 ± 8.95	50.31 ± 8.21
KNN	99.11 ± 1.44	55.6 ± 8.00	51.5 ± 5.58	68.2 ± 6.00	44.35 ± 5.03	62.83 ± 9.32	70.83 ± 11.59
RandomForest	100 ± 0	78.83 ± 6.04	91.12 ± 3.60	94.2 ± 3.34	68.5 ± 5.05	93.69 ± 2.75	96.37 ± 2.94

**Table 8:** The average accuracy (AP%) of various deep learning models in identifying different short-circuit fault types of WT generators.

SENSORC DATASET							
Deep Learning Model	Normal	HI-1	HI-2	HI-3	LI-1	LI-2	LI-3
ResNet-31	99.42 ± 1.31	91.53 ± 4.87	99.23 ± 1.73	99.91 ± 0.21	88.75 ± 7.06	99.57 ± 0.92	100 ± 0
ResNeXt-22	100 ± 0	86.50 ± 7.65	99.11 ± 2.00	99.93 ± 0.16	82.91 ± 7.5	99.59 ± 0.93	98.66 ± 3.00
DenseNet-37	100 ± 0	86.87 ± 5.70	99.15 ± 1.12	99.63 ± 0.82	84.64 ± 8.14	99.56 ± 8.14	98.38 ± 3.62
VggNet-18	100 ± 0	92.24 ± 6.41	99.92 ± 0.15	99.99 ± 0.03	90.36 ± 5.42	99.59 ± 0.93	98.07 ± 4.32
Inception-ResNet-59	100 ± 0	89.99 ± 8.86	99.24 ± 1.14	99.82 ± 0.29	87.9 ± 9.95	99.59 ± 0.93	100 ± 0
FCNet-5	99.13 ± 1.95	97.42 ± 3.33	99.80 ± 0.35	99.91 ± 0.19	98.19 ± 1.64	99.59 ± 0.93	100 ± 0
SENSORA_SENSORV DATASET							
Deep Learning Model	Normal	HI-1	HI-2	HI-3	LI-1	LI-2	LI-3
ResNet-31	100 ± 0	90.04 ± 1.83	98.59 ± 2.46	99.63 ± 0.51	87.15 ± 4.47	98.65 ± 0.78	99.87 ± 0.30
ResNeXt-22	99.94 ± 0.07	86.83 ± 4.22	99.86 ± 0.23	99.96 ± 0.06	73.52 ± 8.77	99.99 ± 0.02	100 ± 0
DenseNet-37	99.99 ± 0.02	86.75 ± 2.82	95.73 ± 2.93	99.36 ± 1.05	77.76 ± 5.64	99.53 ± 0.57	99.94 ± 0.08
VggNet-18	100 ± 0	92.36 ± 3.42	99.23 ± 1.17	99.9 ± 0.22	88.59 ± 4.41	99.72 ± 0.60	100 ± 0
Inception-ResNet-59	100 ± 0	90.77 ± 3.47	98.26 ± 2.02	99.83 ± 0.17	86.88 ± 4.12	99.94 ± 0.07	100 ± 0
FCNet-5	100 ± 0	96.09 ± 1.88	99.77 ± 0.29	99.97 ± 0.07	94.25 ± 3.11	99.6 ± 0.89	100 ± 0

**Table 9:** Performance comparison of different machine learning models.

SENSORC DATASET				
Machine Learning Model	Recall% (macro avg)	ACC%	BlnACC%	mAP%
SVM-LINEAR	90.79 ± 2.39	94.76 ± 1.83	90.79 ± 2.39	91.22 ± 2.24
SVM-RBF	89.97 ± 1.71	94.27 ± 1.15	89.97 ± 1.71	90.34 ± 1.68
SVM-POLY	**91.3 ± 2.25**	**95.00 ± 1.86**	**91.30 ± 2.25**	**91.66 ± 2.12**
Bayes(Gaussian)	66.58 ± 3.29	71.35 ± 3.12	66.58 ± 3.29	67.62 ± 3.36
KNN	73.43 ± 3.15	75.82 ± 4.00	72.42 ± 1.60	74.55 ± 3.08
RandomForest	87.19 ± 1.66	91.88 ± 1.27	87.32 ± 1.65	87.9 ± 1.61
SENSORA_SENSORV DATASET				
Machine Learning Model	Recall% (macro avg)	ACC%	BlnACC%	mAP%
SVM-LINEAR	85.61 ± 1.56	85.81 ± 1.78	85.61 ± 1.56	90.6 ± 1.08
SVM-RBF	**88.09 ± 1.71**	**88.53 ± 1.99**	**88.09 ± 1.71**	**94.27 ± 1.35**
SVM-POLY	87.23 ± 0.76	87.4 ± 0.95	87.23 ± 0.76	91.76 ± 1.07
Bayes(Gaussian)	45.22 ± 1.87	46.57 ± 1.70	45.22 ± 1.87	47.82 ± 2.50
KNN	56.37 ± 3.28	58.79 ± 3.50	56.37 ± 3.28	64.63 ± 3.47
RandomForest	82.88 ± 2.16	83.4 ± 2.39	82.88 ± 2.16	88.96 ± 1.12

The best results are highlighted in **bold**.

**Table 10:** Performance comparison of different deep learning models.

SENSORC DATASET						
Deep Learning Model	Model Complexity		Fault Diagnosis Performance			
	FLOPs	Params	Recall%	ACC%	BlnACC%	mAP%
ResNet-31	5.894 M	244.967 k	96.29 ± 1.75	96.39 ± 1.72	96.29 ± 1.75	96.91 ± 1.96
ResNeXt-22	**58.496 k**	**391.000 B**	95.13 ± 2.50	95.13 ± 2.50	94.97 ± 2.59	95.24 ± 2.61
DenseNet-37	624.064 k	2.278 k	95.29 ± 2.12	95.43 ± 2.07	95.29 ± 2.12	95.46 ± 2.15
VggNet-18	8.532 M	232.807 k	96.86 ± 2.00	96.90 ± 1.91	96.86 ± 2.00	97.17 ± 1.66
Inception-ResNet-59	9.886 M	205.527 k	95.82 ± 2.56	95.94 ± 2.57	95.82 ± 2.56	96.65 ± 2.84
FCNet-5	209.920 k	13.367 k	**98.89 ± 0.92**	**98.89 ± 0.94**	**98.89 ± 0.92**	**99.15 ± 0.85**
SENSORA_SENSORV DATASET						
Deep Learning Model	Model complexity		Fault diagnosis performance			
	FLOPs	Params	Recall%	ACC%	BlnACC%	mAP%
ResNet-31	10.182 M	244.967 k	93.62 ± 1.49	93.74 ± 1.21	93.62 ± 1.49	96.27 ± 0.88
ResNeXt-22	**78.464 k**	**391.000 B**	92.86 ± 0.90	92.75 ± 0.94	92.86 ± 0.90	94.30 ± 1.81
DenseNet-37	1.637 M	2.278 k	92.93 ± 2.08	92.98 ± 2.13	92.93 ± 2.08	94.15 ± 1.31
VggNet-18	12.017 M	232.807 k	95.63 ± 0.91	95.7 ± 0.87	95.63 ± 0.91	97.11 ± 1.07
Inception-ResNet-59	15.004 M	205.527 k	94.82 ± 0.53	94.87 ± 0.51	94.82 ± 0.53	95.97 ± 0.56
FCNet-5	215.552 k	13.367 k	**97.22 ± 1.08**	**97.28 ± 1.05**	**97.22 ± 1.08**	**98.52 ± 0.71**

The best results are highlighted in **bold**.

**Table 11:** Performance ranking of deep learning models based on five-fold cross-validation experiments.

	Model	ResNet-31	ResNeXt-22	DenseNet-37	VggNet-18	Inception-ResNet-59	FCNet-5
K-Fold							
0		4	6	5	1	3	2
1		2	5	4	6	3	1
2		5	3	6	4	2	1
3		4	6	3	2	5	1
4		3	6	4	2	5	1
Average rank		3.6	5.2	4.4	3.0	3.6	1.2

**Table 12:** Comparison with existing methods reported in the literature.

SENSORC DATASET								
Model	Normal%	HI-1%	HI-2%	HI-3%	LI-1%	LI-2%	LI-3%	ACC%
MLP [19]	99.98	73.51	66.00	94.05	58.94	98.89	100	84.48
HOS-SR [65]	98.93	82.46	60.56	73.27	80.93	78.89	81.96	94.44
HOS-Bayes [65]	99.60	62.62	60.56	52.73	60.56	78.89	78.61	91.76
Linear FCN [66]	100	95.60	96.10	96.20	91.00	99.00	97.50	96.68
Hierarchical Linear FCN [66]	100	95.60	95.50	97.80	91.50	100	96.20	96.90
VggNet-18 (Ours)	100	92.24	99.92	**99.99**	90.39	**99.59**	98.07	96.90
ResNet-31 (Ours)	**99.42**	91.53	99.23	99.91	88.75	99.57	**100**	96.39
FCNet-5 (Ours)	99.13	**97.42**	**99.80**	99.91	**98.19**	**99.59**	**100**	**98.89**

The best results are highlighted in **bold**.

**Figure A1:**
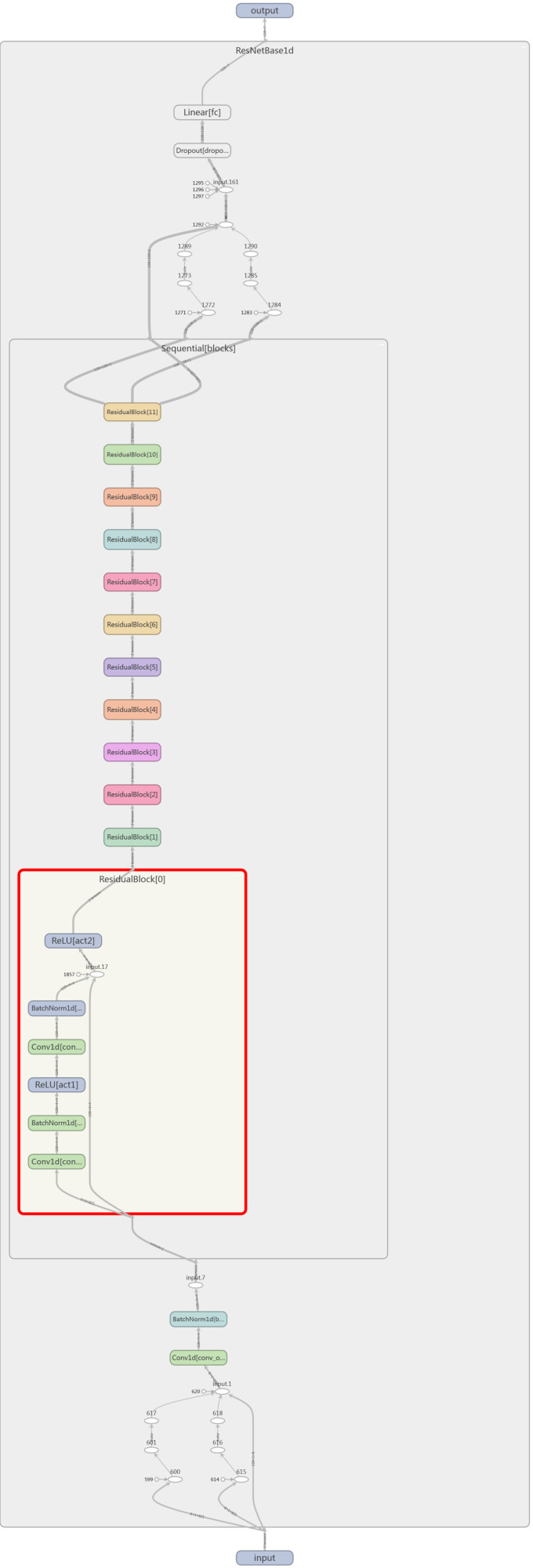
ResNet-31.

**Figure A2:**
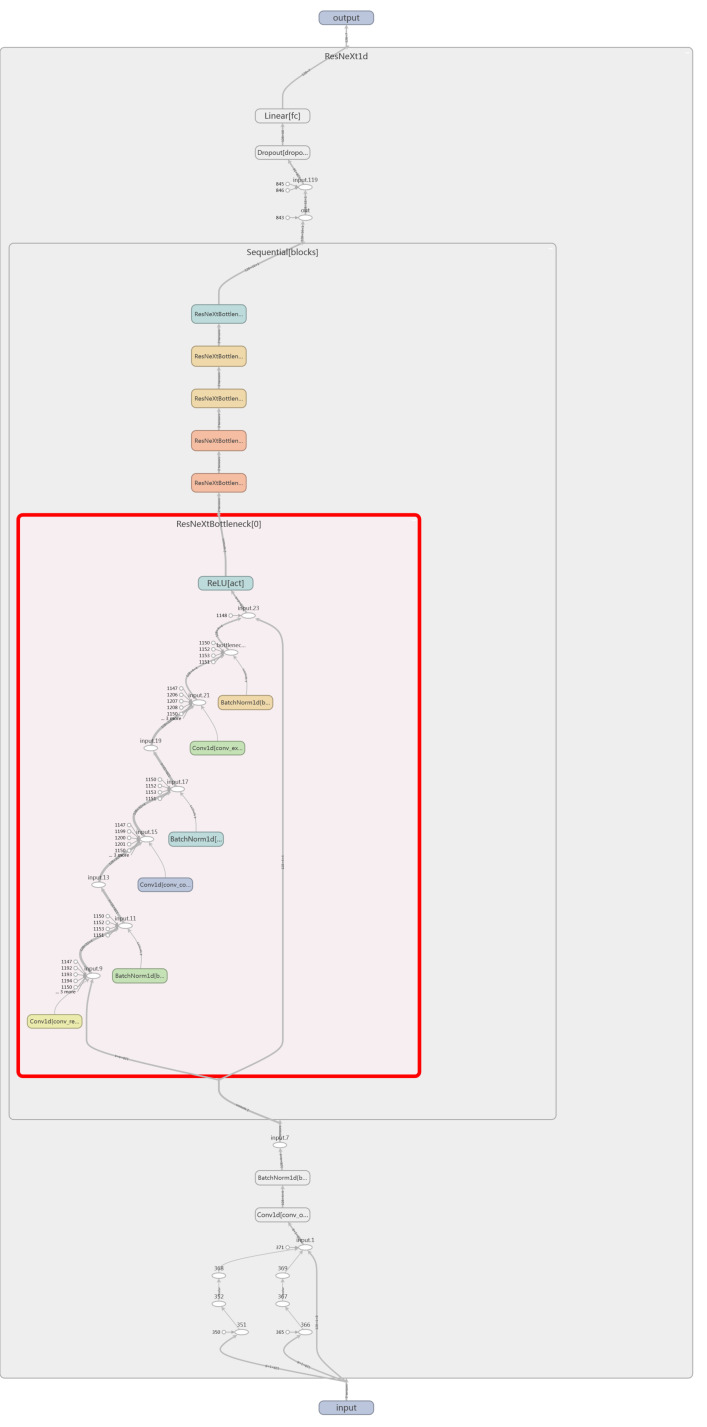
ResNeXt-22.

**Figure A3:**
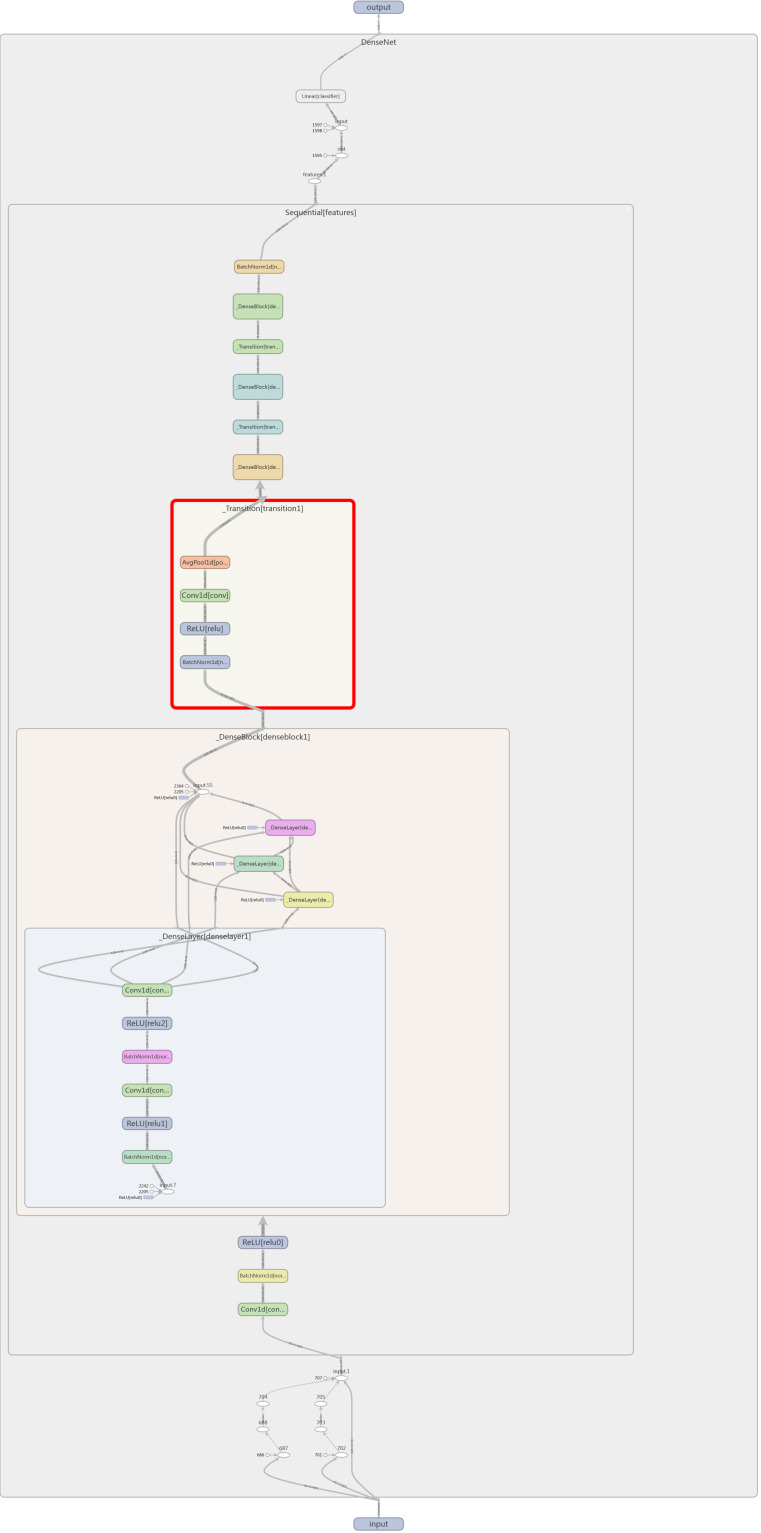
DenseNet-37.

**Figure A4:**
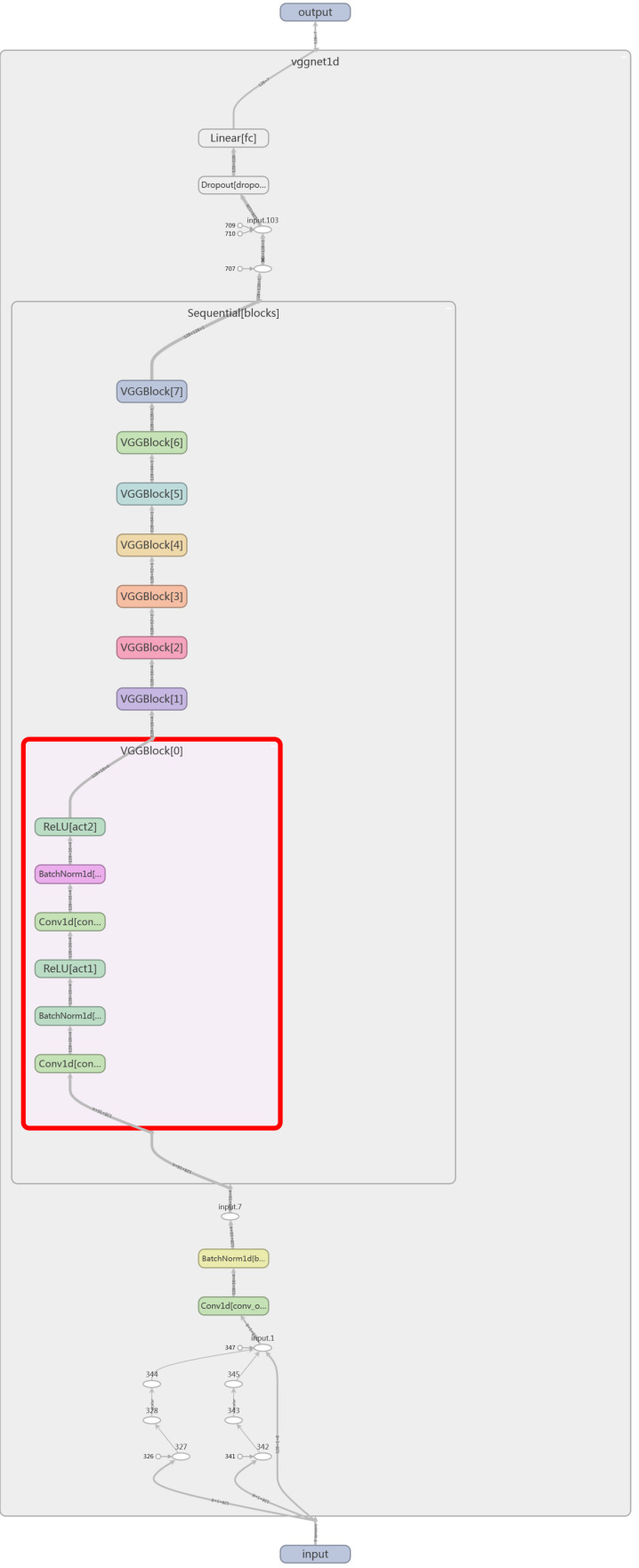
VggNet-18.

**Figure A5:**
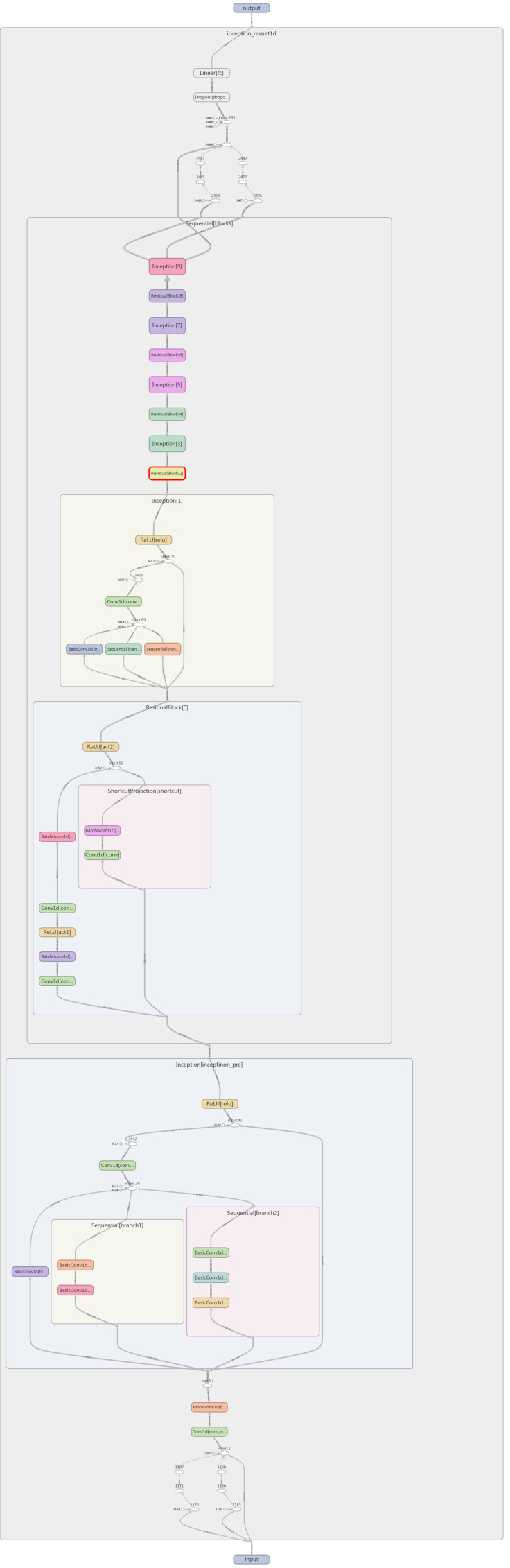
Inception-ResNet-59.

**Figure A6:**
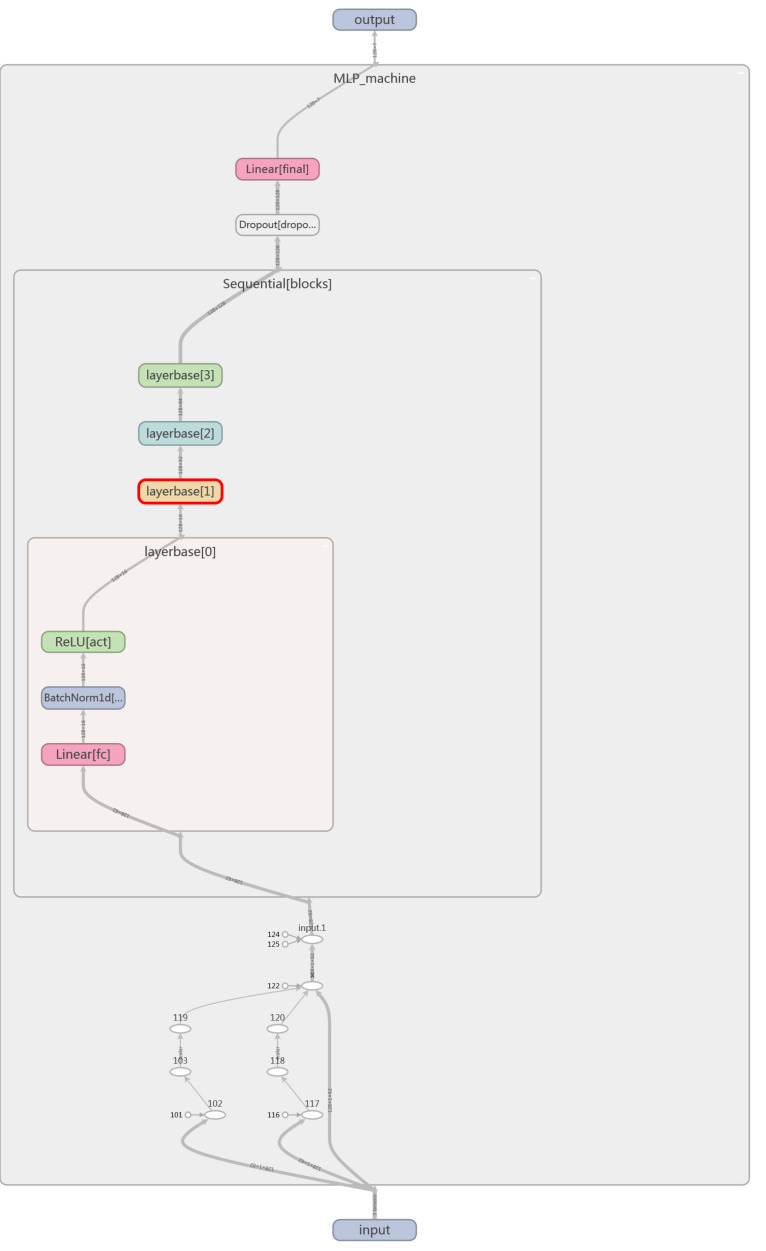
FCNet-5.

## Data Availability

The dataset supporting this study is openly available on GitHub at the following URL: https://github.com/lapisco/Wind_turbine_failure_prediction (accessed on 16 April 2025).

## Appendix A. Comparison of the Hyperparameters of the Model

To clarify our choice of the optimization algorithm, batch size, and initial learning rate, we conducted a comparative experiment to assess the impact of different optimization algorithms and hyperparameter settings on model performance. The VggNet-18 architecture was used for all experiments. The results are presented in Table A1, Table A2, Table A3, Table A4, Table A5 and Table A6 below.

**Table A1:** Comparison of AP% scores of models under different optimization algorithms.

Optimizer	Normal	HI-1	HI-2	HI-3	LI-1	LI-2	LI-3
SGD	**100 ± 0**	82.55 ± 4.74	95.01 ± 5.02	99.38 ± 0.74	76.56 ± 9.00	99.40 ± 0.92	98.07 ± 4.32
Adam	**100 ± 0**	68.12 ± 8.26	77.77 ± 4.27	93.17 ± 2.30	56.94 ± 6.61	99.54 ± 0.90	98.05 ± 4.31
**AdaBound**	**100 ± 0**	**92.24 ± 6.41**	**99.92 ± 0.15**	**99.99 ± 0.03**	**90.36 ± 5.42**	**99.59 ± 0.93**	**98.07 ± 4.32**

The best results are highlighted in **bold**.

**Table A2:** Comparison of model performance under different optimization algorithms.

Optimizer	Recall%	ACC%	BlnACC%	mAP%
SGD	91.95 ± 2.70	92.25 ± 2.47	91.95 ± 2.70	93.00 ± 2.88
Adam	85.11 ± 0.83	85.84 ± 0.86	85.11 ± 0.83	84.79 ± 2.33
**AdaBound**	**96.86 ± 2.00**	**96.90 ± 1.91**	**96.86 ± 2.00**	**97.17 ± 1.66**

The best results are highlighted in **bold**.

**Table A3:** Comparison of AP% scores of models under different Batch sizes.

Batch Size	Normal	HI-1	HI-2	HI-3	LI-1	LI-2	LI-3
8	100 ± 0	89.25 ± 8.02	99.64 ± 0.74	**100 ± 0**	85.31 ± 8.96	**100 ± 0**	**100 ± 0**
**16**	**100 ± 0**	**92.24 ± 6.41**	**99.92 ± 0.15**	99.99 ± 0.03	**90.36 ± 5.42**	99.59 ± 0.93	98.07 ± 4.32
32	100 ± 0	85.86 ± 7.05	98.60 ± 1.03	99.76 ± 0.23	83.54 ± 7.43	99.50 ± 0.90	97.95 ± 4.11

The best results are highlighted in **bold**.

**Table A4:** Comparison of model performance under different Batch sizes.

Batch Size	Recall%	ACC%	BlnACC%	mAP%
8	95.63 ± 1.16	95.65 ± 1.09	95.63 ± 1.16	96.32 ± 2.22
**16**	**96.86 ± 2.00**	**96.90 ± 1.91**	**96.86 ± 2.00**	**97.17 ± 1.66**
32	93.66 ± 2.05	93.88 ± 2.04	93.66 ± 2.05	95.03 ± 2.51

The best results are highlighted in **bold**.

**Table A5:** Comparison of AP% scores of models under different initial learning rates.

Learning Rate	Normal	HI-1	HI-2	HI-3	LI-1	LI-2	LI-3
5 × 10^−3^	**100 ± 0**	89.70 ± 8.77	99.27 ± 1.63	99.97 ± 0.07	89.10 ± 8.62	**99.59 ± 0.93**	**100 ± 0**
**3** **× 10^−3^**	**100 ± 0**	**92.24 ± 6.41**	**99.92 ± 0.15**	**99.99 ± 0.03**	**90.36 ± 5.42**	**99.59 ± 0.93**	98.07 ± 4.32
1 × 10^−3^	99.05 ± 2.12	87.61 ± 7.19	99.04 ± 1.99	99.80 ± 0.37	85.23 ± 13.97	**99.59 ± 0.93**	**100 ± 0**

The best results are highlighted in **bold**.

**Table A6:** Comparison of model performance under different initial learning rates.

Learning Rate	Recall%	ACC%	BlnACC%	mAP%
5 × 10^−3^	95.94 ± 2.44	96.09 ± 2.46	95.94 ± 2.44	96.80 ± 2.77
**3 × 10^−3^**	**96.86 ± 2.00**	**96.90 ± 1.91**	**96.86 ± 2.00**	**97.17 ± 1.66**
1 × 10^−3^	95.35 ± 2.60	95.50 ± 2.59	95.35 ± 2.60	95.76 ± 3.67

The best results are highlighted in **bold**.

## Appendix B. Network Model Structure

We have visualized the architecture of our network models, specifically highlighting the key details of the constructed ResNet-31, ResNeXt-22, DenseNet-37, VggNet-18, FCNet-5, and Inception-ResNet-59.
